# The Bitter Barricading of Prostaglandin Biosynthesis Pathway: Understanding the Molecular Mechanism of Selective Cyclooxygenase-2 Inhibition by Amarogentin, a Secoiridoid Glycoside from *Swertia chirayita*

**DOI:** 10.1371/journal.pone.0090637

**Published:** 2014-03-06

**Authors:** Shantanu Shukla, Khushboo Bafna, Durai Sundar, Sunil S. Thorat

**Affiliations:** 1 Bioresource Database and Bioinformatics Division, Regional Center of Institute of Bioresources and Sustainable Development, Tadong, Gangtok, Sikkim, India; 2 Department of Biochemical Engineering and Biotechnology, Indian Institute of Technology Delhi, Hauz Khas, New Delhi, India; 3 Distributed Information Sub-Centre, Institute of Bioresources and Sustainable Development, Imphal, Manipur, India; Russian Academy of Sciences, Institute for Biological Instrumentation, Russian Federation

## Abstract

*Swertia chirayita*, a medicinal herb inhabiting the challenging terrains and high altitudes of the Himalayas, is a rich source of essential phytochemical isolates. Amarogentin, a bitter secoiridoid glycoside from *S. chirayita*, shows varied activity in several patho-physiological conditions, predominantly in leishmaniasis and carcinogenesis. Experimental analysis has revealed that amarogentin downregulates the cyclooxygenase-2 (COX-2) activity and helps to curtail skin carcinogenesis in mouse models; however, there exists no account on selective inhibition of the inducible cyclooxygenase (COX) isoform by amarogentin. Hence the computer-aided drug discovery methods were used to unravel the COX-2 inhibitory mechanism of amarogentin and to check its selectivity for the inducible isoform over the constitutive one. The generated theoretical models of both isoforms were subjected to molecular docking analysis with amarogentin and twenty-one other Food and Drug Authority (FDA) approved lead molecules. The post-docking binding energy profile of amarogentin was comparable to the binding energy profiles of the FDA approved selective COX-2 inhibitors. Subsequent molecular dynamics simulation analysis delineated the difference in the stability of both complexes, with amarogentin-COX-2 complex being more stable after 40ns simulation. The total binding free energy calculated by MMGBSA for the amarogentin-COX-2 complex was −52.35 KCal/mol against a binding free energy of −8.57 KCal/mol for amarogentin-COX-1 complex, suggesting a possible selective inhibition of the COX-2 protein by the natural inhibitor. Amarogentin achieves this potential selectivity by small, yet significant, structural differences inherent to the binding cavities of the two isoforms. Hypothetically, it might block the entry of the natural substrates in the hydrophobic binding channel of the COX-2, inhibiting the cyclooxygenation step. To sum up briefly, this work highlights the mechanism of the possible selective COX-2 inhibition by amarogentin and endorses the possibility of obtaining efficient, futuristic and targeted therapeutic agents for relieving inflammation and malignancy from this phytochemical source.

## Introduction

The pharmacological journey of curing pain and inflammation has witnessed several *eureka* moments of its own with synthesis of aspirin, in the late nineteenth century, being one of the most notable development. However, the underlying mechanism of action of this wonder drug remained largely elusive for the next seven decades. It was not until 1971 when J. R. Vane and co-workers delineated the mechanistic insights into the action of aspirin and aspirin-like drugs, better known as Nonsteroidal Anti-inflammatory Drugs (NSAIDs), theoretically bolstered by the subsequent experimental findings of Smith and Willis [1]–[3]. The mechanism of aspirin action was centred on the reduction of prostaglandin levels, ringed derivatives of open chain fatty acids, thereby regulating the inflammatory responses mediated by these prostaglandins. The proposed theory was not only capable of explaining the prostaglandin inhibitory mechanisms of NSAIDs, but it also suggested the inherent cause of their attributed side-effects that evidently appeared in the course of treatment when these drugs were administered. Later, it was observed that the NSAIDs act upon the prostaglandin synthesis pathway affecting its production by inhibiting the rate limiting enzyme cyclooxygenase (COX; E.C. 1.14.99.1; later to be known as cyclooxygenase-1 or COX-1) [4]. On the quest to identify better inhibitors of COX, modifications were made to the existing drugs and new analogs were introduced to achieve higher effectiveness with lower side-effect profile. When the development of new leads targeting COX was conceived to reach a saturation point, a second isoform, cyclooxygenases-2 (COX-2), was purified in 1991, independently by the groups of Simmons and Herschman [5], [6]. This new isoform was found to be inducible unlike its constitutive counterpart; thereby, promptly becoming a favourite target for selective COX-2 inhibitors or *coxibs*. The coxibs were different in their mode of action and unlike their antediluvian partners as they targeted specifically the inducible COX-2, with over 50 fold selectivity, without altering the activity of the constitutive isoform COX-1 [7].

At the pathway level, cyclooxygenases convert arachidonic acid (AA) into prostaglandins through a two-step catalytic mechanism; initially oxidising the AA to an intermediate prostaglandin called hydroperoxy endoperoxide (PGG_2_), the cyclooxygenation step, and subsequently reducing it into the hydroxyl endoperoxide (PGH_2_) that serves as the precursor for various prostanoids, the peroxidation step [8]. Belonging to the myeloperoxidase superfamily, cyclooxygenases are homodimeric membrane-bound proteins comprising of three functional domains: the N-terminal epidermal growth factor (EGF)-like domain, the membrane binding domain, and the C-terminal catalytic domain [9], [10]. The catalytic domain harbours the active site which is placed at the end of a long hydrophobic cavity covering the area between the membrane binding domain all the way to the heme group [11]. COX enzymes facilitate the production of prostaglandins that perform a number of colligated physiological processes which may differ with the site of their action. They most prominently promote tissue growth by inhibiting apoptosis, especially in the case of tissue damage repair [12], and enhance the DNA-synthesis mechanism by increasing cell proliferation and cell motility [13]. COX-1 is unremittingly expressed throughout different tissues of the body and is concerned with homeostasis and other housekeeping functions, like maintenance of gastric mucosa, regulating the function of kidneys and platelet aggregation [8], [14]. COX-2, on the contrary, is both constitutive and inducible in nature. It is known to express normally in the tissues of brain and spinal cord where it determines the inflammatory pain response, mediated by interleukin-1β [15], and possibly regulates the process of fever development [16]. It can also be induced across the various tissues of the body in response to specific inflammatory and tumourogenic signals. The two isoforms differ broadly in terms of their gene locus, gene size, number of exons, functional mRNA size, spectrum of substrate utilisation, residual changes in specific positions in the protein active site and side pocket, and, above all, the way they influence certain physiological process [17]. The molecular weight, however, of both COX-1 and COX-2 proteins is ∼68 kD and both isoforms have the ability to perform similar tasks of catalysing the rate limiting step in conversion of essential fatty acids to desired prostaglandins. The intriguing difference in the genetic makeover and the structure-function integrity of these isoforms is not very well understood as it is observed that when COX-1 is knocked-out, COX-2 is normally synthesized by the cell and it carries out the general function of COX-1 without triggering inflammation or hyperproliferation [18], [19].

Recent reports have suggested a negative correlation between the administration of NSAIDs and carcinogenic proliferations of different tissue-types, like breast, oesophagus [20], prostate [21], gastrointestinal tract [22] and most significant of the large intestine (colon and rectum) [23], hinting towards an inherent link between inflammation and malignancy. In accordance to these findings, it was clear that COX-2 was in some manner related to tumour progression. The molecular investigation unearthed a nexus between different tumorigenic signalling cascades and their association with the inducible activity of COX-2. It was observed that expression of COX-2 mRNA was enhanced, through the protein kinase C pathway (PKC) and RAS-signalling cascade, in the presence of tumour promoting signals, like cytokines and growth factors [24]. Additionally, in the case of colon cancer progression, COX-2 significantly controlled the apoptotic pathway by upregulating BCL-2 through Ras-mitogen activated protein kinase (Ras-MAPK) /ERK pathways, thus helping the tumour cells to evade apoptosis [25]. These findings buttressed the argument that controlling the COX-2 activity was directly linked to a reduction in the cellular malignancy and therefore the COX-2 was an important target for both anti-inflammatory and anti-cancer therapy.

In contrast to the synthetic drugs, natural products have been used since antiquity to cure pain and inflammation. Initial concoctions for curing pain were prepared from the barks of the willow tree (*Salix sp.*) by Hippocrates and dates as early as 400 B.C. [26]. It was later identified by Raffaele Piria, in the year 1838, that the active principle of the essential oil extracted from willow tree was associated with its salicylic acid content [27]. Subsequently, a major breakthrough was achieved in the year 1897 when Felix Hoffman, an employee with the Bayer & Co. acetylated the natural salicylates to form acetyl salicylic acid, better to be known as aspirin [28]. This naturally derived drug became a huge success story. There were little revelations from the arena of natural products and soon the tide shifted largely towards the synthetically derived compounds. However, the advent of the twenty-first century was greeted with a burgeoning world population, with new anomalies and a voracious appetite for drug consumption. There was an inherent need to introduce new therapeutic lead molecules with innovative action and higher effectiveness profile. This forced, both, the pharmaceutical industry and the research community to subsequently look for new and better formulations thus, retracting the course of identification of novel compounds back towards the nature.

Since then, several Plant Derived Molecular Entities (PDMEs) have become an important component of present pharmaceutical industries with various formulations currently under clinical trial [29]. It is worth mentioning that a wide range of PDMEs show significant anti-inflammatory properties by targeting arachidonic acid pathway (extensively review by [30]). Studies have indicated that isolates possessing anti-inflammatory activity also own significant anticancer properties. Among the most significant ones, PDMEs like curcumin, isolated from turmeric, epigallocatechin gallate (EGCG), a polyphenol from green tea, and resveratrol from grapes, have a strong anti-inflammatory property and are proven anticancer agents, achieving this by suppressing the NF-κB signalling and, in turn, regulating the expression and activation of COX-2 [31]. Similar activities have been observed with ajoene, a phytochemical isolate from garlic, which showed an enhanced activity against COX-2, IC_50_ of 3.4 μM, by targeting the mRNA expression [32]. Natural products, therefore, are an important source of lead molecules which can be innovatively manipulated to bring about better formulations with increased activity.

*Swertia chirayita*, a medicinal herb growing at an altitudinal range of 1200 to 3200 m around the Himalayan and sub-Himalayan terrains of Pakistan, India, Nepal, Bhutan, and Tibet, has been used for centuries by the traditional healers to cure malarial fever and arthritic pain. *S. chirayita* contains a variety of phytochemicals which encompass alkaloids, xanthones, phenols, terpinoids, flavonoids, iridoids and secoiridoids [33]. Concoctions prepared from *S. chirayita* have important therapeutic implications, like as a hepatoprotective, an antipyretic, an anthelmintic, a hypoglycaemic, and an anticancer agent. Recent reports have suggested that treatment with a crude extract of *S. chirayita* containing mangiferin, amarogentin, and swertiamarine show a positive correlation with the reduction in blood sugar [34]. It has also been observed that the chloroform and butanol soluble fractions of the methanolic extract of the plant are effective against the hepatotoxicity induced by carbon tetrachloride and paracetamol, evidently proving its hepatoprotective properties [35], [36]. *S. chirayita* harbours great anti-cancer properties, as it was found that, both crude and purified, extracts were potent anticarcinogen and activators of apoptosis which they achieved by upregulating the phase II detoxifying enzymes [37]. Therefore, this plant tenders itself as an excellent bioresource for new lead compound identification which could be effective for targeted therapies.

**Figure 1:**
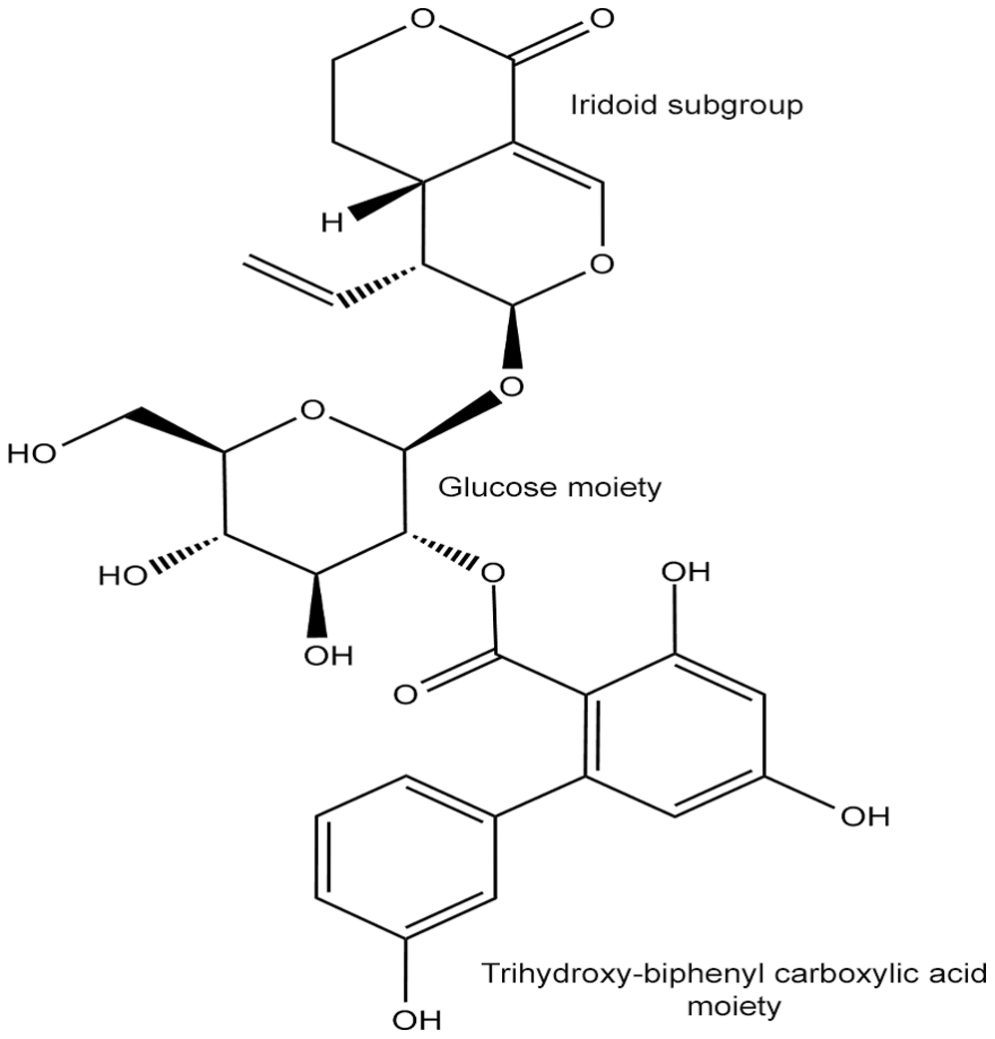
Structure of Amarogentin, a secoiridoid glycoside from *Swertia chirayita*. Amarogentin consists of three essential subgroups, the iridoid group, the glucose moiety and the biphenyl-triol rings.

Among the various chemical constituents present in *S. chirayita*, amarogentin [Figure 1], a bitter secoiridoid glycoside, is a proven anti-lieshmanial agent [38] and an activator of human bitter taste receptor hTAS2R50 [39]. The compound has a unique property of functioning as a targeted entity for site directed therapies, as well as for undirected ones [40]. Recent studies have addressed that amarogentin can act against liver carcinogenesis by regulating the activity of G1/S cell cycle checkpoint kinase in carbon tetrachloride (CCl_4_)/N-nitrosodiethylamine (NDEA) -induced liver carcinogenesis in mouse models [41]. In another interesting experiment Saha and co-workers reported that in case of mouse skin carcinogenesis model, amarogentin curtailed the tumour progression by downlegulating the expression of COX-2 and agonistically influencing the apoptotic mechanism governed by Caspase-3, with an IC_50_ of 0.5 mg, though, the maximum activity was observed at a concentration of 0.2 mg/mouse, much lower than the estimated IC_50_ [42]. The study provided a strong *in vitro* experimental proof of potential anti-carcinogenic activity of amarogentin, which it achieved by abating the hyperproliferative capacity of COX-2. Though, this study did not report any deleterious gastrointestinal ulceration in the murine models used, there was also no mention of the selectivity of amarogentin for the inducible isoform over the constitutive one. So, to support the fact that amarogentin can actually be regarded as a potential inhibitor of COX-2, it has to be specifically selective for the induced isoform.

We, therefore, took-up the task to understand the molecular mechanism of amarogentin action on both COX-1 and COX-2, which can suggest a possible selectivity for the induced isoform over the constitutive one. To achieve this objective, we applied the computer-aided drug discovery methods like molecular modelling, molecular docking and molecular dynamic simulation. Initially, the structures of both isoforms were modelled and after quality assurance, they were minimized for docking. Comparative docking analysis of amarogentin was performed against the selected FDA approved lead molecules to get a comparative overview of its expected activity. The docked structures of amarogentin and three selective COX inhibitors, that is celecoxib, valdecoxib and etoricoxib, with lowest binding energies were subjected to a molecular dynamic simulation analysis to observe the stability of the protein- ligand complex. Finally, the binding free energy calculation was performed on the post-simulation complexes to validate the attributed energy values for ligand, protein and the protein-ligand complex. The larger focus of the present study, however, was to highlight amarogentin as possible selective inhibitors of COX-2, enabling further research and development of novel drugs derived from this PDME.

## Methods

### Homology modelling of the target proteins

The protein sequences for Human COX-1 [Uniprot ID: P23219] and COX-2 [Uniprot ID: P35354] were retrieved from the Uniprot Knowledgebase [43]. Subsequently, these sequences were subjected to a pairwise alignment using the Basic Local Alignment Search Tool for protein sequences (BLASTp) [44] against the Protein Data Bank (PDB) [45] to find suitable template structures for both the proteins. In this regard, the crystal structure of *Ovis aeris* COX-1 [PDB Id: 1DIY], at a resolution of 3.00Å, with 94% identity and 92% query coverage, and the crystal structure of *Mus musculus* COX-2 [PDB Id: 1CVU], at a resolution of 2.40Å, with an overall identity of 88% and a query coverage of 91%, were selected as templates for COX-1 and COX-2, respectively. The residue numbering, used here, is different from the residue numbering of the selected PDB templates, for instance, residue numbering in COX-1 model numbering is one minus the residue number in 1DIY (for example, Arg 120 in 1DIY is Arg 119 in the COX-1 modelled protein), whereas, the residue number of COX-2 model is given by fourteen minus the residue number of 1CVU (for example, Arg120 in 1CVU is Arg106 in COX-2-model). Selection of template, in both the cases, was categorically scrutinized on the basis of various parametric indicators like percentage of similarity between the target and the template sequences, e-value of sequences, query coverage and number of insertions and deletions. The final template sequences, showing the best overall alignment score, were chosen individually for both the proteins. The amino acid sequences of the selected individual templates were aligned to the amino acid sequences of the two respective target protein using MultAlin [46]. Prior to the modelling, the alignment file (alignment.ali), and template structure file (template.pdb) were assembled in a working directory. The template structures were then used to generate three dimensional structures of the Human COX-1 and COX-2 proteins using the stored files and *model-ligand.py* script of program MODELLER 9.11 [47]. Ten models were generated for each of the two proteins with the substrate AA overlaid in its corresponding position. The generated models were assessed using DOPE score (*assess_dope.py*) and Molpdf score (*assess_ga314.py*), generated by MODELLER 9.11, along with the Verify3D score [48] and Errat score [49]. Finally, the best model was selected based on the result of combined assessment analysis of above mentioned scoring functions and was subsequently stabilised using and molecular dynamics simulation.

### Stabilisation of the modelled proteins

The GROMACS 4.5.5 [50] package (single precision) was used to perform molecular dynamics (MD) simulation of the modelled proteins and all the protein-ligand complexes. The topologies for the ligands were generated using the ProDrg 2.5 server [51]. The protein topologies were prepared using the GROMOS96 43a1 force field, inherent to the Gromacs 4.5.5 package. Both modelled proteins were centred in a cubic box 0.80 nm from the box edge and the box was solvated with three point water model SPC. To neutralise the charged protein appropriate counter ions were added to the system. The solvated electroneutral system was relaxed through Steepest Descent energy minimization. Periodic boundary conditions were chosen to ensure that the protein remains well within the box while exploring the conformational space. The systems were equilibrated under the isothermal-isochoric ensemble followed by isothermal-isobaric ensemble, each for a time frame of 100ps. The production molecular dynamics (MD) simulation of 15ns and 10ns was performed for COX-1 and COX-2, respectively, with a time step of 2fs. Modified Berendsen thermostat V-rescale was used for temperature coupling and Parrinello-Rahman pressure coupling was used. Reference temperature was kept as 300K with a coupling constant 0.1ps. Bond lengths were constrained using the procedure LINCS. Long-range electrostatics was evaluated using the Particle Mesh Ewald (PME) summation. The output of coordinates, trajectory, velocities and energies were recorded at every 2ps. Production MD was run till the final 5ns time frame was stable for both the protein simulations. To check the stability of the structures different analyses, like Root Mean Square Deviation (RMSD) of the protein backbone (g_rms), Root Mean Square Fluctuations (RMSF) of the individual residues of the protein molecule (g_rmsf), Radius of gyration (Rg) of the entire protein molecule (g_gyrate), intra-protein hydrogen bond interactions analysis (g_hbond), were carried out using GROMACS 4.5.5. The lowest energy frame was extracted from the last 5ns stabilised region of the MD simulation trajectory. Subsequently, the ions and solvent molecules were removed from the extracted frame and this structure was further used for molecular docking.

### Molecular Docking

Molecular docking is an important tool used for scoring various poses of the ligand-receptor conformations and ranking those poses according to their overall stability, in terms of their binding energies. Docking algorithms can give insight into the molecular mechanism of inhibition of the target by the small molecule, by predicting the binding energy of their interaction, orientation of ligand within the receptor binding cavity, and the effect of the protein environment on the charge distribution on the ligand [52]. We, therefore, performed virtual molecular docking on amarogentin and 21 Food and Drug Association (FDA) approved small molecules, selective and non-selective, (listed in **Table 1**) against the cyclooxygenases. The reason we did a comparative docking assessment of amarogentin against already approved leads were for two specific reasons, (i) we wanted to check whether the binding pattern of amarogentin was closer to selective or to the non-selective inhibitors of COX, and if the binding was significantly closer to selective inhibitors, then (ii) to access its comparative performance with the highly selective inhibitors.

**Table 1:** AutoDock binding energy values and H-bond forming residues of the lead molecules.

	COX-1		COX-2	
Ligands	AutoDock Binding Energy	H-bond Forming Residues	AutoDock Binding Energy	H-bond Forming Residues
Amarogentin	−7.67	Arg119, Ser352	−10.32	Tyr371, Ser516, Arg106, Met508
Aspirin	−5.91	Ser529, Tyr347	−6.03	Ser516
Celecoxib	−8.37	Tyr384, Ser529, Tyr347	−8.6	Ser516, Ser339
Diclofenac	−7.19	Tyr347	−7.45	Ser516, Tyr371
Etodolac	−7.87	Ser529	−7.19	Ser339
Etoricoxib	−8.8	Tyr347	−10.41	Arg499
Flurbiprofen	8.76	Arg119 (2 H Bonds)	−8.61	Arg106, Tyr341
Ibuprofen	−7.81	Arg119 (2 H Bonds)	−7.34	Trp373, Phe367
Indometacin	−5.12	Tyr354, Ile522	−8.35	Tyr341
Ketoprofen	−9.04	Tyr384, Arg119	−8.81	Ser516, Ala513, Met508
Ketorolac	−8.08	Arg119 (2 H Bonds)	−8.45	Trp373
Lumiracoxib	−7.05	Arg119 (2 H Bonds)	−8.17	Ser516
Meloxicam	−8.13	None	−10.29	Tyr371 (2 H bonds)
Nabumetone	−8.13	Arg375	−8.67	Tyr371
Naproxen	−7.88	Arg375	−8.24	Tyr371, Arg106
Nimesulide	−8.69	Ala201, Tyr347, Ser529	−9.51	Tyr371, Ser516
Parecoxib	−10.73	Ser529	−11.67	Ser339, Val335
Piroxicam	−8.29	Ser529	−9.6	Ser516, Ser339, Ala513, Val 509
Rofecoxib	−9.45	Tyr347	−9.72	Tyr371, Trp373
Sulindac	−6.75	Tyr347	−9.87	Phe367
Tolmetin	−7.92	Arg119 (2 H bonds)	−8.09	Trp373, Ser516
Valdecoxib	−8.8	Ser529, Phe528, Tyr347	−9.51	Ser339 (2 H bonds), Val335

Docking prediction of high scoring poses of different COX inhibitors and their corresponding binding energy values was compared with the binding free energy of amarogentin. Subsequently, the H-bonding residues were also analysed for the stability of the docking poses.

Therefore, to predict the interactions of candidate ligand with their biomacromolecular target, AutoDockTools (ADT) v1.5.4 and AutoDock v4.2 [53] were used. The stabilised protein structures extracted from molecular dynamics simulation trajectory were used for docking studies. AA coordinates were removed from both the stabilised complexes and only the target protein coordinates were saved. The receptor and ligand coordinate files were prepared using ADT in AutoDock specific coordinate file format, termed PDBQT. The receptor preparation included adding all hydrogens to the macromolecule, calculating partial atomic charges from the Gasteiger method and assigning AutoDock4 (AD4) atom types. The 3D coordinates of ligand molecules were downloaded from the free chemical database ChemSpider [54] and prepared using ADT. The standard docking procedure was followed for a rigid protein and flexible ligand whose torsion angles were identified. Affinity maps for all atom types present, as well as electrostatic maps were computed with a grid of 50, 52, and 52 points in *x*, *y*, and *z* directions, respectively, and with a grid spacing of 0.375 Å. The Lamarckian genetic algorithm method was employed for 15 long docking simulation runs, while all other parameters were left as default. Using ADT the docking results were clustered based on conformational similarity and the protein-ligand interactions were visualised and saved in the PDB file format.

### Molecular Dynamics Simulation of COX-Amarogentin complexes

A concurrent MD simulation was carried out for amarogentin-COX-1 and amarogentin-COX-2 complexes respectively, to address the behaviour of the ligand and protein in close vicinity. Similar parametric values, as described for the protein stabilisation dynamics, were applied to the ligand-protein complex run and all the protein-ligand complex simulations were carried out using GROMACS 4.5.5. Topologies for the ligand molecules were prepared using ProDrg 2.5 server. The docked complexes of amarogentin with COX-1 and COX-2, having the lowest binding scores, were simulated for 40ns to study the binding pattern and stability of ligand amarogentin within the active-site cavity of the two proteins, respectively. Additionally, we performed MD simulations for celecoxib-COX-2, valdecoxib-COX-2 and etoricoxib-COX-2 complexes, each for 20ns, using the same parameters as used in the case of amarogentin-COX-1 and amarogentin-COX-2 complexes. Index groups were created, using make_ndx script in GROMACS 4.5.5, to facilitate the selection of specified groups for further analysis. Analyses of the MD simulations, like RMSD, RMSF, R_g_, intra-protein hydrogen bonding, protein-ligand hydrogen bonding, hydrogen bonding between critical residues and the ligand molecule, were accomplished using various GROMACS analysis programs and GRACE plotting tool (http://plasma-gate.weizmann.ac.il/Grace/).

### Binding Free Energy Analysis

A binding free energy analysis was carried out, for both the protein–ligand complexes (COX-1 and COX-2 with amarogentin) and three FDA approved selective molecules, celecoxib, etoricoxib and valdecoxib, using the MMGBSA (Molecular Mechanics-Generalized Born Surface Area) methods implemented in the AMBER Tools v12 (script *mm_pbsa.pl* by Ross Walker & Thomas Steinbrecher). Snapshots of COX-amarogentin complexes were extracted from the last 20ns of the MD trajectories at an interval of every 100ps. For other ligands equal number of snapshots were extracted from the stable section of RMSD trajectory. Interaction energies and solvation free energies were calculated individually for each of the complex, receptor and ligand to obtain an estimate of binding free energy, respectively, in each case using the MMGBSA protocol as described in [55]. The final calculation excluded the entropy contribution to binding and, therefore, the free energy of binding would be an approximation of binding energy and can be used to compare relative ligand binding affinities of the complexes [56], [57]. For the MMGBSA calculation explicit water is replaced by solvent continuum enabling direct calculation of binding free energy[58], [59]. Dielectric constant for implicit solvent was set to 80. The Generalized Born (GB) methodology [60] was applied to solve the polar contribution, using the parameterization as provided in [61]. Linear Combination of Pairwise Overlap (LCPO) method [62] was used to compute solvent accessible surface area. Individual binding energies for the complex, the protein, and the ligand molecule were calculated and the total binding free energy of the system, given by the sum of the individual binding free energies of these entities, was calculated. The difference between the binding energies of the complex, the free-protein, and free-ligand gives the totalbinding free energy value of the system, which is an indicative of the stability of the protein – ligand complex, is represented by the binding free energy term ΔG_bind_.

Thermodynamic cycles employed for the MMGBSA calculations are represented below.



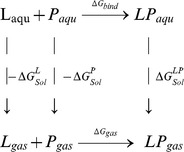



Where L is ligand, P is protein, LP is protein-ligand complex, _aqu_ is aqueous phase and _gas_ is gas phase. For each snapshot, binding free energy was calculated using the following equations:
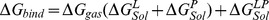
(1)


Where, ***▵G_bind_*** is the total binding free energy of the system, which is calculated as the difference of the ***▵G_gas_***_**,**_ the interaction energies of the ligand and protein in the gas phase, and the solvation free energies of the ligand, the protein and the complex, represented by ***▵G^L^_Sol_***_,_ ***▵G^P^_Sol_***, and ***▵G^LP^_Sol_*** respectively. 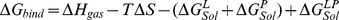
(2)


Here, ***▵H_gas_*** represents the enthalpy term in the gas phase and ***T▵S*** is the conformational binding entropy.



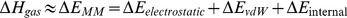
(3)


The gas phase MM energy, given by the term ***▵E_MM_*** is the sum of contributing terms of the electrostatic energy, ***▵E_electrostatic_***, the van der Waals energy, ***▵E_vdw_*** and internal energies of the bonds, angles and dihedrals, ***▵E_internal_***



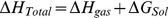
(4)


The total enthalpy term ***▵H_Total_*** is the summation of the gas phase enthalpy and salvation free energy. And the solvation free energy ***▵G_Sol_*** is the sum of the electrostatic or polar solvation free energy ***▵G_polar_*** and the non-polar solvation free energy, ***▵G_non-polar_*** terms.



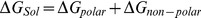
(5)


## Results and Discussion

### Homology modelling of the target proteins

The BLASTp results of the sequences, from both proteins, yielded multiple hits, but selective crystal structures containing the natural substrate, AA, bound to COX-1 and COX-2 were narrowed down upon. Selections of the templates were also influenced by their overall alignment score with the target sequences. These selections were done in order to, initially, understand the binding of the natural substrate in the active site channel, giving a better idea of blocking its access to the binding pocket, and subsequently, to keep the shape of the channel intact through the entire course of the analysis. Further, the alignment of the target and the template residues indicated large chunks of conserved regions between the target and the template sequences, especially that of the active site forming residues, [Figure S1]. The above results are in strict accordance with the findings made by Chandrasekharan and Simmons [9] and endorse the fact that orthologs of both COX isoforms are conserved in vertebrates.

Supported by the initial findings, we generated 10 theoretical models, each, for COX-1 and COX-2, based on the MultAlin sequence alignment and model validation was carried out to identify the most favoured model. The best selected model of COX-1 showed an overall DOPE score of −69291, with a Verify3D score of 83% and an ERRAT quality factor analysis of ∼70. Similarly, the best selected model of COX-2 indicated a DOPE score of −67654, a Verify3D score of 80.50% and a quality factor score of 74.47 from the ERRAT server. Since the structures gave a low quality factor score, a further refinement was carried out by removing the heme group from the close proximity of the active site and by trimming the N- and C-terminal residues of the model which did not have an implicated effect on the active site symmetry, as described by Price and Jorgensen [63]. The quality of the processed models significantly improved after the refinement as it was observed that the Verify3D score and quality factor analysis score from ERRAT surged to 89.17% and to ∼73, respectively, for the COX-1 model. Similar scoring improvements were observed in the COX-2 model, with an ERRAT quality analysis score of ∼77 and a Verify3D score of around 91%. The overall structural integrity of the modelled proteins was further substantiated by a Ramachandran plot analysis which indicated nearly 90% residues in the most favoured region and 0.2% in the disallowed region for the COX-1 model, and had ∼93% residues in the most favoured region and no residues in the disallowed region for the COX-2 model. Residues corresponding to the binding cavity were present in the most favoured region, hinting towards no disorientation of the active site residues lining the cavity. Subsequent stabilisation of the protein models was carried out using molecular dynamics simulation to address the discrepancy surrounding the Ramachandran plot values of COX-1 and COX-2 and to standardize the structures for molecular docking.

### Stabilisation of the modelled proteins

**Figure 2:**
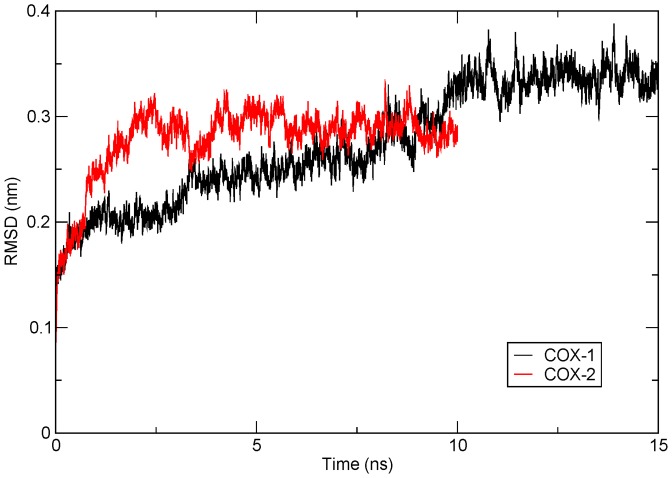
RMSD of the backbone of COX-1 and COX-2 modelled proteins after stabilisation. Protein backbone RMSD of COX-1 model, over a time frame of 15ns (in black), shows stability in the last 5ns time frame, deviating about 0.35nm from the native structure whereas, protein backbone RMSD of COX-2 model, for 10ns (in red), achieved stability after 5ns and maintained till the final 10ns, with an average deviation of about 0.3nm. The plot has been generated using the GRACE plotting tool.

To impart stability to both modelled proteins, they were subjected to a MD simulation for 15ns and 10ns for COX-1 and COX-2, respectively. Presence of AA prevented the shrinkage of the binding cavity during the entire MD simulation. The overall protein stability was scored on various parametric features of the MD run, like the comparative assessment of RMSD, RMSF, and Rg of the initial and final frames of the MD run; and an appraisal of the intra-protein hydrogen bonding to check its overall stability in the course of the simulation. Analyses were conducted only after observing the atomic fluctuations of the protein in a constant x-axis, for a time frame of about 5ns, indicating a fairly stabilised model which can be used for systemic analysis. In this regard, the RMSD of all backbone atoms of the protein were observed to deviate up to 0.35nm, in case of COX-1, and up to 0.30nm, in case of COX-2 [Figure 2]. The COX-1 model attained stability after 10ns of MD run and maintained it through the final 5ns. On the contrary, COX-2 stabilised after the initial 5ns run and remained stable in the final 5ns. From the above results, it can be inferred that during the course of protein folding, the backbone lost its initial flexibility as the tertiary structure moved closer to a stable conformation indicating that the modelled protein did not correspond to the most stable conformation.

**Figure 3:**
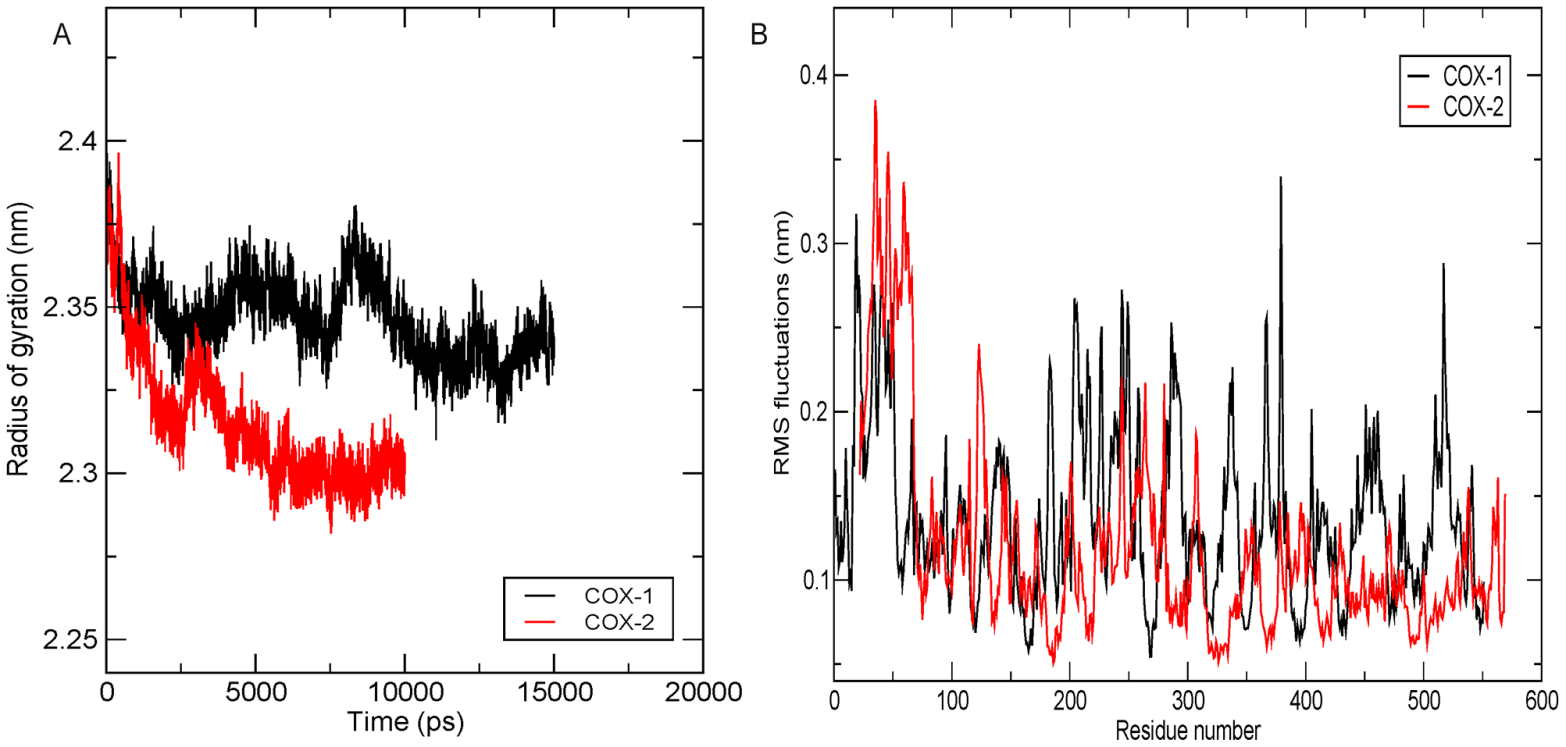
Protein stability assessment in terms of Rg and RMSF. (A) The overall movement in the protein, with respect to the initial frame, is calculated using Rg plot. Rg plot for COX-1 (in black) showed an average deviation of 2.34nm and an average deviation of 2.30nm for COX-2 (in red). (B) Residue fluctuations were observed using the RMSF plot and the overall fluctuation was found to be marginal for both the proteins, with a maximum deviation occurring along the terminal residues and the loop region. The active site residues showed intactness for both COX-1 (black) and COX-2 (red) protein models with very low observed fluctuations. The plots have been generated using the GRACE plotting tool.

Another degree measure of the structural stability in MD simulation is the Rg. Rg sums-up the structural integrity of the model which is a direct implication of the inter-atomic distances of the protein residues in due course of the simulation. Both the models showed a fairly consistent Rg in the final 5ns time frame, with COX-1 showing a Rg of 2.34nm and COX-2 of a 2.30nm [Figure 3A]. This improved consistency in the Rg suggested a gradual stability attained by the structure in course of their folding, without compromising on the atomic compactness of their overall tertiary structures.

Similar inferences were drawn by comparing the RMSF of the two structures. Except for the terminal regions, which tend to show more fluctuation than the core residues, the RMSF for both COX-1 and COX-2 was observed to be around 0.2nm, implying a fair stability of the protein core over the course of simulation [Figure 3B]. The fluctuations observed in COX-1 and COX-2, around the core region, can be attributed to the presence of a large number of loops in the tertiary structure of both the isoforms. Since the loops are observed to have higher variability, it is known that the mean fluctuations of these regions are greater than the other secondary structures which are, generally, kept intact by the presence of H-bonds.

Analysis of intra-protein hydrogen bonding of both modelled proteins [Figure S2] suggested a gradual stability after initial fluctuations, similar as observed in the case of their respective Rg plots. COX-1 made, on an average, 390 intra-protein hydrogen bonds (H-bonds), with an initial increase in the H-bond numbers till 7ns time frame and stabilising thereafter. Similarly, it was observed that the COX-2 protein model depicted an increased H-bond number up to the initial 5ns time frame but gradually stabilised for last 4ns time frame, with an average H-bond number around 410. There was an observed correlation between the initial increase in the RMSD and the intra-protein H-bonding of the two models, both of which gradually reached stability in the latter half of their respective MD runs. The initial deviations in the intra-protein H-bonding, the RMSD and the Rg, clearly suggest that the modelled protein did not, initially, qualify in terms of stability but with the progression of the simulation it reached a minimum-energy-maximum-stability phase. There was very little or no deviation observed in the models after they attained stability and was, therefore, regarded fit for further computational analysis.

### Molecular Docking

Amarogentin showed a high binding affinity for COX-2, with a binding energy of −10.32kcal/mol, against a binding energy of −7.67 kcal/mol for COX-1. Binding energy of amarogentin clustered with the binding energies of highly selective COX-2 inhibitors, like etoricoxib (−10.41 kcal/mol) and parecoxib (−11.67 kcal/mol). The reason for such, marginal yet characteristic, discrepancy in the binding energy values can be understood in terms of the difference in the binding site residues of the two isoforms. Among the most important differences, as highlighted by [8], [14], [64], the transition of Ile523 (Ile522 in the model COX-1) in COX-1 to Val523 (Val509 in COX-2 model) in COX-2 holds the key to selectivity. The Ile side chain is bulky and hence, sterically hinders the binding of larger compounds, like the selective COX-2 inhibitors. On the contrary, Val has a smaller side chain, having one methyl group less than the Ile side chain, providing just enough space for the bulky cyclic groups of the selective inhibitors to occupy.

**Figure 4:**
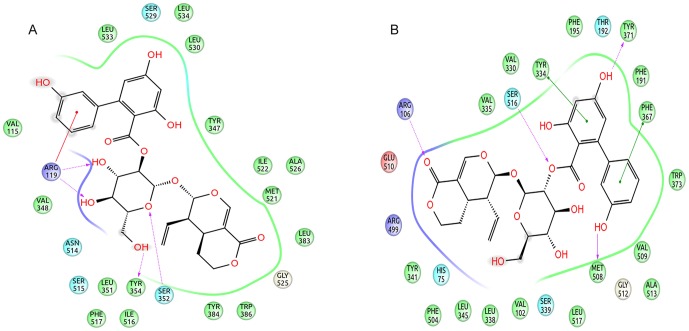
2D representation of the docking pose of amarogentin inside the hydrophobic cavity of COX. (A) The docking pose of amarogentin inside the binding cavity of COX-1 shows that besides forming several van der Waals interactions, amarogentin forms four H-bonds, two with Arg119 and one with Ser352. (B) A total of five H-bonds were also observed in the docked amarogentin-COX-2 complex, one each with Arg106, Ser516, Tyr371, and Met508. Amarogentin formed van der Waals interaction with a several residues along the hydrophobic channel. (Legend: green spheres represent hydrophobic residues; cyan spheres represent residues with polar side chains; red spheres and purple spheres show negatively and positively charged amino acid residues, respectively; solid red lines denote cation- π interaction; dotted purple lines indicate hydrogen bonds with side-chain atoms, with the direction of arrow denoting the acceptor atom; solid green lines indicate π-π interactions; and the grey spheres surrounding the atoms indicate that the atoms are exposed to solvent). The illustrations have been generated using Schrödinger Maestro open-source visualisation package.

Structure of amarogentin can be segregated into three subunits, with the iridoid (tetrahydropyranone) group and the trihydroxy-biphenyl carboxylic acid (TBCA) moiety at two opposite ends clumped together by a glucose molecule in the middle. In COX-1, amarogentin made a total of two H-bonds, one between Arg119 and the hydroxyl side chain of glucose, and the other between the Ser352 and the oxygen atom of the pyranose ring [Figure 4A and Figure S3A]. A cation-π interaction was observed between Arg119 and the phenol ring of the TBCA moiety. The ligand also formed hydrophobic contacts with approximately 20 residues across the entire stretch of the hydrophobic channel. It however did not engage with the Ser529 in forming an H-bond, reason being the distance of over 3.5Å. This observation implicated towards a steric hindrance caused by the presence of Ile, not allowing the biphenyl group of the TBCA moiety to accommodate closer to the catalytic site residue in COX-1. Interactions with COX-2 yielded five H-bonds, two with Arg106, both with the pyran oxygen of the iridoid group, one each with Ser516, with the glycosidic oxygen, Tyr371, with the resorcinol moiety of the biphenyl sub-group, and Met508, with the phenol ring of the TBCA [Figure 4B and Figure S3B]. The structure was further stabilised with the help of twelve hydrophobic residues and three polar residues making van der Waals contacts with the ligand. There were two coulombic interactions made by Glu510 and Arg499, providing an anchor towards the mouth of the channel. Quite uniquely, there were two π-π interactions, one with the phenyl group of the Tyr334 side chain and the benzoic acid ring of the TBCA, and the other between the benzene ring of Phe367 and the phenol group of the TBCA. The high binding energy of amarogentin, in case of COX-2, can be understood in terms of its above mentioned interaction profile, giving it a much stable appearance when compared to its binding pose with the COX-1. The Ile/Val transition at the position 523 (522 in the modelled COX-1 and 509 in the modelled COX-2) accounted for a marginal increase in the cavity space, enough for the biphenyl ring of amarogentin to accommodate without much steric resistance. The binding pattern of amarogentin, with the COX isoforms, was similar to the binding of the selective COX-2 inhibitors, which bind specifically around the catalytic site inside the hydrophobic channel. The final analysis of the two ligand-protein complexes therefore, suggested that both interactions were fairly stable, with amarogentin looking more comfortable inside the binding cavity of the COX-2. The complexes were thereafter, subjected to a molecular dynamics simulation analysis to evaluate their ligand-protein interaction and the overall stability of the complex, with respect to time.

As a clear understanding of the actual binding pattern of any ligand cannot, directly, be inferred from its best docking pose hence, the efficiency of the docking algorithm becomes important in cases where a comparative assessment is being carried out between the established and the novel leads. In this accord, we searched for existing crystal structures of selective and non-selective inhibitors bound to COX on the PDB database and retrieved ten crystal structure records, five each for COX-1 and COX-2 [Table S3]. Subsequently, we analysed their binding and orientation and compared it to the predicted docking poses. Due to the absence of crystal structures for the majority of the above listed ligands, the smaller sample size of co-crystallised lead molecules can possibly result in a bias while addressing the overall efficiency of the AutoDock, which recently was identified to be efficient in correlating the docking poses to the experimental data with a comparable efficiency to that of the commercial docking algorithms, like Gold and Glide [65]. We observed that docked poses were fairly similar to the crystal orientation for the available crystal structures with the RMSD of the docked poses to their crystal structure orientations ranging between 0.15Å to 1.13Å, implying towards the high efficiency of the docking algorithm. Therefore, to support the docking predictions, a comparative analysis of the actual stability of both ligand-receptor complexes was carried out using long MD simulations.

### Molecular Dynamics Simulation of COX-Inhibitor complexes

The stability of amarogentin and the three coxibs inside the binding cavity of the target was reviewed using a 40ns and 20ns MD simulation, respectively. Since the change in the ligand and protein RMSDs, over the course of the entire MD run, can predict the overall stability of their complex, we generated separate RMSD plots for the ligands and the COX isoforms to analyse their overall behaviour in the course of the simulation.

**Figure 5:**
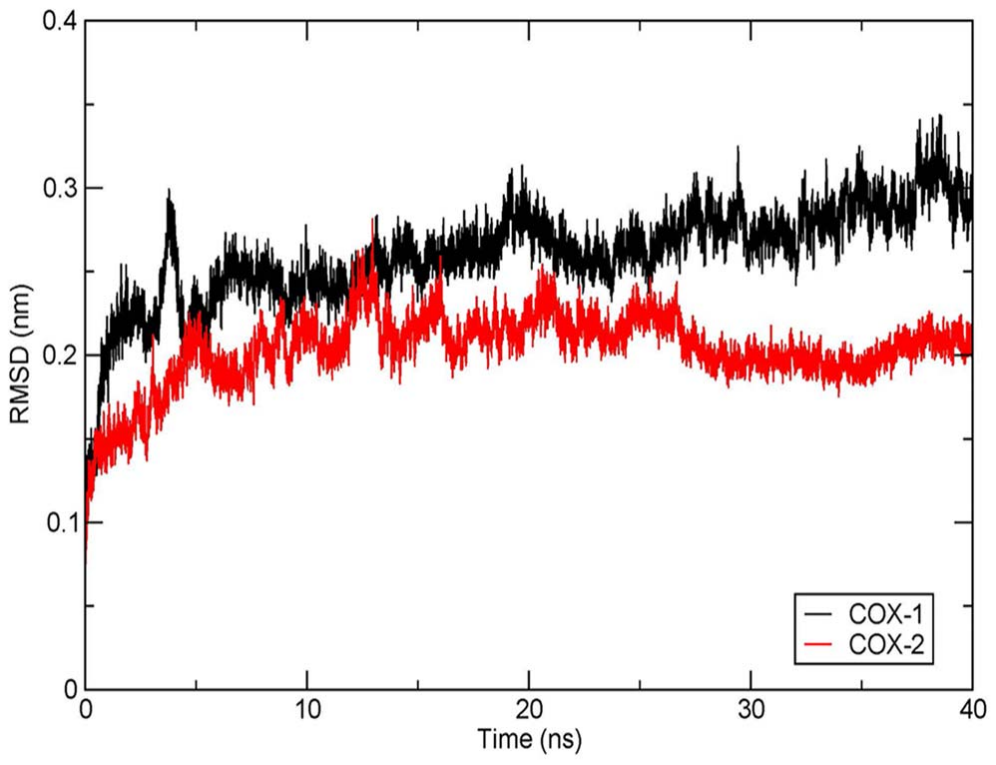
RMSD of amarogentin-COX complexes for 40ns time frame. RMSD of amarogentin-COX-1 complex (black) shows an elevation in the deviation from the initial structure, reaching around 0.3nm for the final 40ns frame. The RMSD of amarogentin-COX-2 complex (red) showed initial deviations but attained stability at 15ns and remained so till the final 40ns time frame with an RMSD of 0.18nm. The plots have been generated using the GRACE plotting tool.

While observing the amarogentin-COX complex it was observed that the protein RMSD [Figure 5] highlighted that COX-1 did not attain a stable conformation for a long stretch of the entire 40ns run, deviating by almost 0.3nm from the native structure. The COX-1 RMSD initially rose to 0.3nm at 3ns, came down marginally to 0.25nm at approximately 4.5ns and kept on elevating thereafter, till the final 40ns. Protein RMSD of COX-2 gave indications of an initial increase in the RMSD, reaching ∼2.3nm at around 5ns time frame and then attaining significant stability around 15ns. Surprisingly, the RMSD of COX-2 further dipped to about 0.18nm at around 28ns and remained stable, fluctuating in a very narrow range till the final 40ns. It was observed in context to the above results that the deviation in the RMSD of the COX-1 was comprehensively larger than that of the COX-2, suggesting the formation of a possible stable complex of the amarogentin with the latter. Subsequent analysis of the RMSD of the amarogentin gave a completely different picture about its stability in the two protein complexes. It was observed in the case of COX-1that amarogentin initially deviated up to ∼0.3nm around 7ns time frame, thereafter stabilising at an average RMSD of ∼0.23nm for the rest 40ns [Figure S5A]. To investigate this seemingly unusual phenomenon, we analysed the frames just after the ligand attained stability and observed that the amarogentin had completely disoriented from its initial position and that it had slightly shifted from the constricted upper channel towards the lower end of the binding pocket, where that conformation was marginally preferred to the previous one. On the other hand, amarogentin indicated a correlated stability with COX-2, as it was observed that after reaching a peak value of ∼0.22nm around the 7ns time frame, the RMSD of the ligand remained stable across next 20ns MD run, with an average RMSD of around 0.2nm [Figure S5B]. As observed with the RMSD of the COX-2, the RMSD of the amarogentin came down to ∼1.8nm around 30ns time frame, and remained the same for the final 10ns of the MD run. This correlated stability of the amarogentin and the COX-2 hinted towards a better inhibition when compared to the inhibition of the COX-1 by the amarogentin.

**Figure 6:**
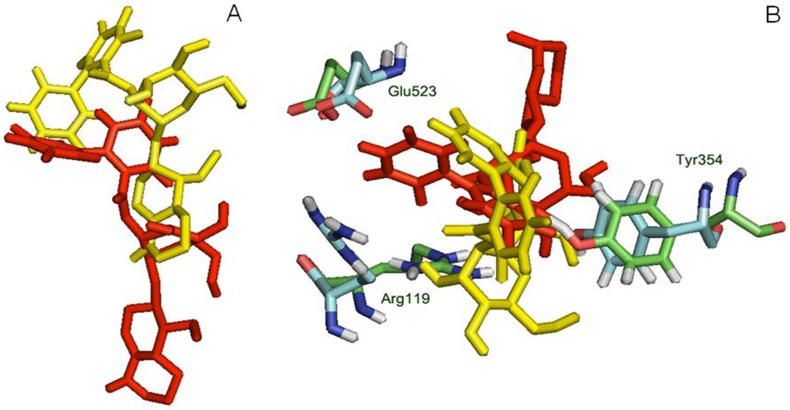
Interaction of amarogentin with COX-1 based on its position at 0ns and 40ns. (A) Interaction of amarogentin with channel gate forming residues, at 0ns (red) and 40ns (yellow). The movement in the position and angle of the residue, at 0ns (green) and 40ns (cyan), can differentiate their deviations and suggest a movement of amarogentin outside the channel breaching the channel gate. (B) Position of amarogentin at 0ns (red) and at 40ns (yellow) clearly indicates a complete shift in its orientation, in the course of the simulation. The figure was generated using the PyMol molecular visualisation tool.

**Figure 7:**
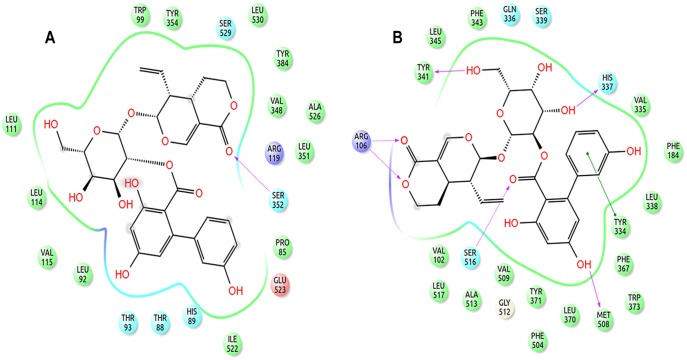
2D representation of the binding pose of amarogentin inside the hydrophobic cavity of COX isoforms after 40ns simulation. (A) Amarogentin formed only one H-bond with Ser352 in the COX-1 hydrophobic cavity. However, atoms which formed H-bonds after docking were present as van der Waal contacts. (B) Amarogentin made a total of six hydrogen bonds and was positioned up in the channel making the pose look more stable after simulation. Legends are same as expressed in Figure 4. The illustrations have been generated using Schrödinger Maestro open-source visualisation package.

**Figure 8:**
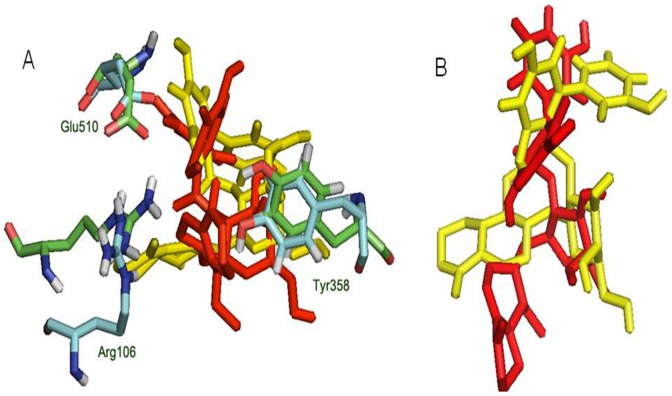
Interaction of amarogentin with COX-2 based on its position at 0ns and 40ns. (A) Interaction of amarogentin with channel gate forming residues, at 0ns (red) and 40ns (yellow). There is comparatively less deviation in the side chains of channel gate residues at 0ns (green) and at 40ns (cyan). (B) Change in the position of amarogentin at 0ns (red) and at 40ns (yellow) indicate a marginal deviation in its structure at 40ns from the native 0ns structure. The illustrations were generated using the PyMol molecular visualisation tool.

The difference in the RMSD of protein and ligand, through the entire 40ns frame, was subsequently compared to the change in structural orientation of the ligand in the binding cavity of both proteins. The 40ns frame of both ligand-protein complexes demonstrated significant difference in their individual stability. The amarogentin completely disoriented from its original position in the COX-1 binding cavity [Figure 6A], partially breaching the binding cavity gate [Figure 6B], forming a single H-bond with Ser352 [Figure 7A and Figure S4A]**.** The amarogentin-COX-2 complex attained further stability, in terms of structural orientation of the ligand, with the amarogentin forming a total of six H-bond interactions, two with Arg106 and one each with Tyr341, His337, Met508 and with Ser516 [Figure 7B and Figure S4B]. Above observations indicate that the amarogentin was not able to form desired H-bond interactions with the active site residues of COX-1, the reason being a constricted channel sterically inhibiting a favourable binding, rendering the ligand unstable and thus, changing its orientation by retracing its course towards the binding channel gate [Figure 8]. On the contrary, the reasonable stability of the amarogentin in the COX-2 binding cavity can be attributed to the fact that the smaller side chain of the Val, present near the catalytic site, provided space for the bulky biphenyl moiety of the TBCA to sit, thereby enabling a stronger binding. The difference in the structural stability is, eventually, possible from the superimposition of the initial and final frames of both the complexes. Therefore, the above results entail a possible selectivity profile of the amarogentin for the inducible isoform. MD runs, of 20ns each for celecoxib, valdecoxib and etoricoxib bound to COX-2, were also carried out to account for the binding free energy analysis, which was used to facilitate a comparative view of the possible inhibitory property of amarogentin in context to the established FDA approved compounds. The MD runs in case of amarogentin-COX complexes were extended upto 40ns only to observe their relative stability over the course of simulation, which could shed light on the possible mechanism of selectivity, whereas that was not the objective in case of MD simulations pertaining to the coxibs as they are well established selective COX-2 inhibitors.

### Binding Free Energy Analysis

To estimate the overall binding free energy of the ligand, protein and their complex, so as to correlate it to the extent of their overall stabilisation, we performed MMGBSA analysis. Though both MMPBSA and MMGBSA can be used to calcualate the binding free energy of a ligand-protein system, we have addressed the binding energy in terms of the latter, as it is reportedly more efficient for analysing binding free energy of the MD simulations [66]. The hydrophobic contribution was expressed in terms of solvation free energy, represented by ΔG_non-polar_. The ΔG_non-polar_ values for amarogentin-COX-1 and amarogentin-COX-2 complexes were found to be −7.53 KCal/mol and −7.81 KCal/mol respectively, suggesting a fairly equal hydrophobic contribution for both complexes. This is also in accordance with the MD results, indicating relatively equal number of van der Waals interactions formed by both proteins with the ligand molecule. Another variable contributing to the solvation free energy is the non-bonded electrostatic energy, depicted by ΔG_polar_, whose values were determined to be 27.99KCal/mol and 57.91KCal/mol, for amarogentin-COX-1complex and amarogentin-COX-2 complex, respectively, hinting a swing towards the electrostatic contribution rather than hydrophobic one to stabilise the complexes, subsequently, higher in case of COX-2. To address the total non-polar and polar contributions to the solvation, ΔG_sol_ scores were compared and it was observed to be 20.45KCal/mol for amarogentin-COX-1complex and 50.11KCal/mol for amarogentin-COX-2 complex. ΔG_sol_ values are in agreement with the high ΔG_polar_ score for COX-2 complex, significantly higher than that of COX-1 complex due to greater electrostatic interaction energy contribution. ΔG_Ele_ sums-up the overall electrostatic energies from solvation free energy and the molecular mechanics (MM) electrostatic contributions (ΔE_electrostatic_). The ΔG_GB_ values were observed to be 28.37KCal/mol for amarogentin- COX-1 complex, and 31.11KCal/mol for amarogentin-COX-2 complex. In general, electrostatic interactions in proteins arise due to the presence of anionic amino acids, like aspartic acid, and cationic amino acids, like lysine. When such residues are present in close vicinity of the ligand molecule, they exert a coulombic force at a shorter distance. On examination of the interactions made by amarogentin with COX-1 and COX-2 it was identified that while the latter formed multiple electrostatic interactions, the former failed to contact any positive or negative residues.

The calculated values of the ΔG_bind_ for amarogentin-COX-1 complex was determined to be −8.57KCal/mol, whereas, for amarogentin-COX-2 complex was found to be −52.35KCal/mol. The discrepancy in the ΔG_bind_ values for both complexes implies that the interaction between amarogentin and COX-1 is less favourable in terms of the overall energy supporting this interaction, whereas, the ΔG_bind_ value for amarogentin-COX-2 complex predicts the possibility of a favourable interaction. Inclusion of the entropy term might optimize the real time equivalent of the binding free energy individualistically but for understanding selectivity upon comparison of ΔG_bind_ it might not turn out to be insightful. However, such large divergence in the overall binding free energy value can bolster the possibility that amarogentin can possibly be a selective inhibitor of the inducible COX isoform. Further to support our findings we did a comparative MMGBSA of three FDA approved COX-2 selective inhibitors. The comparison between the calculated binding free energy values and the experimentally derived values is shown in Table 2. The experimental values were derived from the IC50 values [67]. From this table, it is evident that the compounds show linear relationship between the experimentally derived binding free energy and calculated binding free energy. It was also observed that amarogentin has the lowest calculated binding free energy, which suggest it to be a possible selective COX-2 inhibitor. The input files used for the final interaction energy calculations are provided as Supporting Information zip file.

**Table 2:** Comparison of calculated Binding free energies for COX-2 selective drugs with known experimental data.

Ligand	ΔE_electrostatic_	ΔE_vdw_	ΔE_internal_	ΔG_gas_	ΔG_polar_	ΔG_non-polar_	ΔG_sol_	ΔG_Ele_ a	ΔG_bind_	ΔG_exp_ b
Amarogentin	−26.80	−75.66	0.00	−102.46	57.91	−7.61	50.11	31.11	−52.35	−
Celecoxib	−15.51	−51.71	0.00	−67.23	31.47	−4.66	26.81	15.96	−40.41	−10.01
Valdecoxib	−10.82	−43.68	0.00	−54.51	29.67	−3.86	25.81	18.85	−28.70	−8.31
Etoricoxib	−13.48	−59.52	0.00	−73.00	52.28	−6.28	46.01	38.80	−27.00	−8.17

a ΔG_Ele_  =  ΔEelectrostatic + ΔGpolar.

b ΔG_exp_  =  −RTln(IC_50_).

### Overall Interaction of amarogentin with COX-1 and COX-2

The overall stability of the ligand is proportionally linked with its activity, which is in turn influenced by factors, such as ligand orientation within the receptor binding pocket, change in the conformation of the binding site residues with the change in the ligand symmetry, and formation of bonded and/or non-bonded interaction with the residues lining the binding cavity. Similarly, the protein structure is also stabilised by both bonded and non-bonded interaction. The essential bonded interactions stabilising a protein molecule include the peptide bonds and the disulphide linkages. The peptide bonds are formed between the carboxyl group and the amino group of subsequent amino acids. Disulphide linkages are observed when two cysteine residues come close enough to allow electron pairing between two adjacent sulphur atoms. The three dimensional structure of the protein, however, is mainly maintained by the presence of non-bonded interactions which predominantly include the H-bonds, the electrostatic interactions and the van der Waals interactions. H-bonds are formed between molecules containing polar side chains, that is, either hydrogen or oxygen as a side chain residue. Electrostatic interactions result due the presence of charged amino acid residues, like the negatively charged aspartic acid and the positively charged arginine residues, across a very short distance. Some non-bonded interactions, like π-π and cation-π interactions, occur less frequently. A π-π interaction occurs due to the presence of two aromatic rings placed close to each other while the cation-π interaction occurs between a positively charged residue and an aromatic ring. The aggregated sum of all bonded and non-bonded interactions exerted on a molecule, at any time *t*, is known as the applied force field on that molecule. The force field is a time function as all bonded and non-bonded interactions change with respect to time and it fluctuates collaterally with the change in the symmetry of protein-ligand complex. These factors often determine the total binding free energy of the ligand-receptor interaction and are used as a parameter to evaluate the stability of the overall complex. Therefore, to completely appreciate the extent of ligand binding and the stability of both complexes, it is necessary to account for the subtle changes in the values of force field parameters over time.

**Figure 9:**
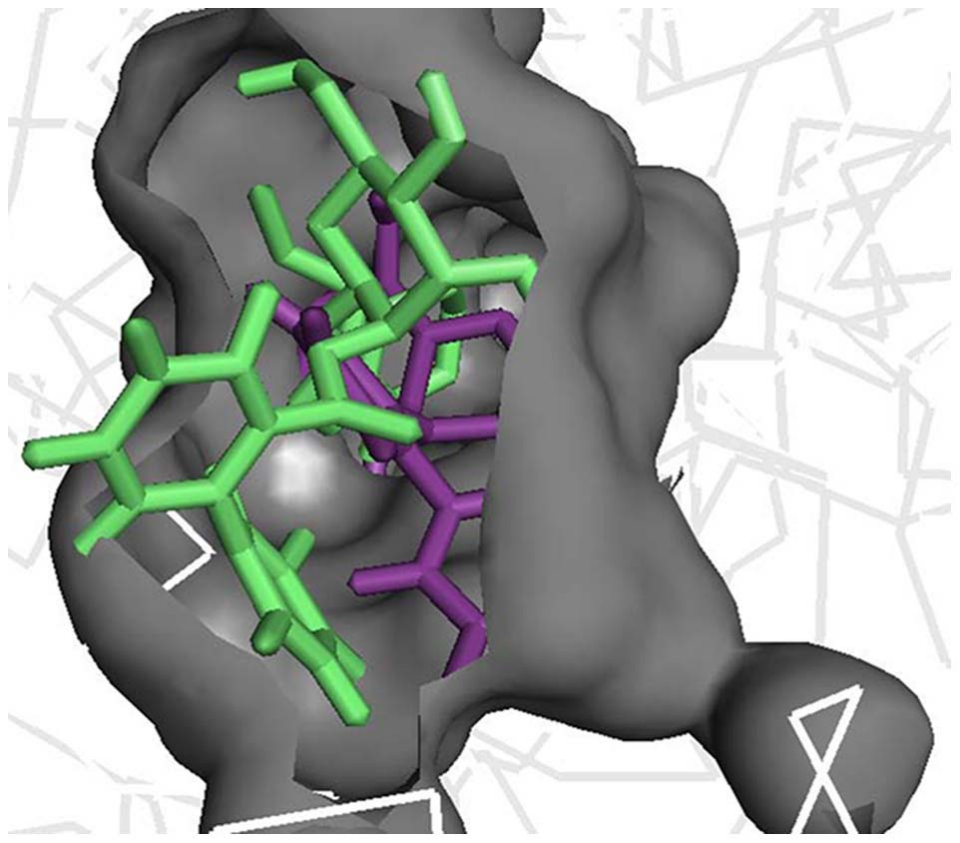
Movement of amarogentin inside the COX-1 binding cavity. Amarogentin after 40ns (green) simulation shows a clear movement outside the binding channel (grey) with respect to its initial 0ns frame (purple), indicating that the complex may not be stable in nature. The figure was generated using the PyMol molecular visualisation tool.

Initially, the docking results of amarogentin with COX-1 and COX-2 gave a vague picture of its binding mode inside the hydrophobic channel and its possible selectivity for COX-2 as both proteins were observed to form an almost equal number of non-bonded contacts with the ligand molecule after docking. As the ligand was bound inside the hydrophobic binding channel, it formed the majority of its non-bonded interaction with hydrophobic and very few with polar residues, in both COX isoforms. There were, however, substantial differences between the bindings of the ligand molecule inside the two protein cavities. As indicated in earlier, the TBCA moiety formed van der Waals interaction with the catalytic site residue Ser529 of COX-1, though it failed to make any H-bond interaction with the same. The phenol ring of the TBCA moiety was partially stabilised by the van der Waals interactions with Val115 and a cation-π interaction of the phenol group with Arg119 side chain. The iridoid subgroup entered deeper in the binding cavity and was surrounded by hydrophobic residues, drawing it to form van der Waals interactions. The glucose molecule acted as an anchor to possibly stabilise the entire molecule, giving it some sort of symmetry. It formed one H-bond each with Arg119 (2.30Å) and Ser352 (2.32Å), acting as an acceptor for both. After 40ns MD run, results indicated the presence of only one H-bond between the iridoid group and Ser352 (1.52 Å). The ligand was also engaged towards forming electrostatic interactions with Glu523 and Arg119. The fall in the number of H-bonds indicated a shear loss in the overall stability of the molecule inside the protein cavity. Amarogentin being a bulky molecule cannot withstand constrictions and steric clashes with the surrounding residues. This was observed after the final 40ns MD run as amarogentin completely changed its orientation, inside the binding cavity of COX-1 and slowly moved towards the cavity gates, breaching them in the process. There was huge difference observed in the initial and final orientation of the gate forming residues Arg119, Glu523 and Tyr354. The movement of amarogentin, partially, outside the binding cavity of COX-1 [Figure 9], few non-bonded interactions after the 40ns MD simulation, and a comparatively higher binding free energy value are all indicative of the very fact that amarogentin-COX-1 complex may not be very stable and the ligand possibly cannot inhibit the protein cyclooxygenation step.

On the contrary, amarogentin demonstrated a better binding mode with the residues lining the hydrophobic cavity of COX-2, supported by the results obtained from docking and subsequent MD simulation analyses. Amarogentin maintained a high H-bond index with COX-2, starting with five H-bonds after docking to six after the final 40ns MD run, suggesting an increased stability of the ligand inside the channel. On close examination of the ligand-protein complex, we observed that amarogentin made two H-bonds with Arg106 (1.85 and 1.93Å), and single H-bonds with Ser516 (1.48Å), Tyr341 (2.34Å), His337 (2.14Å) and Met508 (1.97Å), keeping it well within the hydrophobic channel. Equally high number of van der Waals interactions around the ligand molecule, especially around the TBCA and the iridoid moieties were recorded. Contrasting these values with the values obtained after docking, where amarogentin formed five H-bonds with Arg106 (1.90Å and 2.42Å), Ser516 (2.20Å), Met508 (2.16Å), and Tyr371 (1.95Å), we observe a steady increase in the stability of the ligand. The H-bond distances have reduced significantly in the four conserved interactions and the addition of two more interaction further balances the overall orientation of the structure. The residues which formed H-bonds after docking but failed to do so after the simulation did, however, form close hydrophobic contacts, suggesting a minor deviation in the structural orientation of the ligand. Among the two π-π interactions observed after docking, one with Tyr334 was conserved, adding further to the total stability of the ligand. Analysis of the channel gate forming residues, before and after the simulation suggested no deleterious change in the symmetry of the side chains of these residues. Therefore, subtle variations in the structural organization of the binding channel can bring about a great deal of difference in terms of the selective ligand binding.

In this context, amarogentin showed a binding mode similar to that of the selective inhibitors. The biphenyl ring of the TBCA moiety positioned itself near the active site residue and formed an H-bond with Ser516, acting as an acceptor group by pulling the hydrogen from the terminal hydroxyl group of Ser516. The glucose molecule acts as a donor group while forming H-bonds with the terminal phenol ring in Tyr341 and with the imidazole ring in His337. The iridoid group chokes the channel by forming two H-bond interactions with terminal hydroxyl groups of Arg106 side chain, thus, not allowing the natural substrate to enter inside the cavity. These interactions possibly contribute to a lower binding free energy profile of amarogentin-COX-2 complex, making it a favourable complex. The overall interaction profile of both COX-1 and COX-2, with amarogentin, has been listed in Table 3 **.** Therefore, above evidences enlighten the possible selective mechanism of amarogentin action on COX-2, sparing the constitutive COX-1 in the process. The mechanism of action of amarogentin can be similar to the selective COX-2 inhibitors, which might be attributed to the fact that amarogentin can also utilise the subtle differences in the residual organisation in the binding cavity of the two COX isoforms.

**Table 3:** Overall Non-bonded Interaction profile of amarogentin with COX-1 and COX-2.

	COX-1 After Docking	COX-1 After Simulation	COX-2 After Docking	COX-2 After Simulation
**Hydrogen Bond**	Arg119, Ser352	Ser352	Arg106 (2 bonds), Ser516, Tyr371, Met508	Arg106 ( 2 bonds), Ser516, Met508, Tyr341, His337
**π-π Interaction**	None	None	Tyr334, Phe367	Tyr334
**Cation-π Interaction**	Arg119	None	None	None
**Electrostatic Interaction**	None	Glu523, Arg119	Glu510, Arg499	None
**van der Waals Interaction**	Leu533, Ser529, Leu534, Leu530, Tyr347, Ile522, Ala526, Met521, Leu383, Gly525, Trp386, Tyr384, Ile516, Phe517, Leu351, Ser515, Asn514, and Val384	Leu111, Trp99, Tyr354, Ser529, Leu530, Tyr384, Val348, Ala526, Leu351, Pro85, Ile522, His89, Thr88, Thr93, Leu92, Val115, and Leu114	Val355, Val330, Phe195, Thr192, Phe191, Trp373, Val509, Ala513, Gly512, Leu517, Ser339, Val102, Leu338, Leu345, Phe504, His75, and Tyr371	Leu345, Phe343, Gln336, Ser339, Val335, Phe184, Leu338, Phe367, Trp373, Leu370, Phe504, Tyr371, Gly572, Val509, Ala513, Leu517, and Val102

The total interaction profile of amarogentin covers the H-bond interactions, electrostatic interactions, π-π interactions, cation-π interaction, and the van der Waals interactions.

## Summary

PDMEs have long been serving as precursors for various modern therapeutic concoctions. Several medicinal plants, with immense potential to provide new lead compounds, are often not very well appreciated. These plants have been a part of the ethnomedicinal practice of local communities for several centuries; however, they fail to make any significant global impact. *Swertia chirayita*, a Himalayan medicinal plant, has been known to contain certain constituents which play significant role in curing multiple disorders. Amarogentin, a bitter secoridoid isolate from *S. chirayita*, is reported to have implications in different patho-physiological ailments and inhibition of COX-2 in the mouse skin carcinogenesis model. However, its inhibitory mechanism against the inducible COX isoform is not very well understood. In order to establish the drug-likelihood of amarogentin, it has to be selective in its action, that is, acting on the induced isoform while sparing the constitutive one. This work, therefore, proposes possible selective inhibitory mechanism of amarogentin against COX-2. We observed that the stability of amarogentin-COX-2 complex was par exceeding the stability of amarogentin-COX-1 complex, thus, highlighting its selective profile for the induced isoform. So far, there has been no report suggesting a selective inhibition of COX-2 by a secoiridoid glycoside. This work, therefore, delineates the possibility of finding an efficient and selective COX-2 inhibitor from this phytochemical class, subject to further exploration.

## Supporting Information

Figure S1 **Pairwise sequence alignment of COX-1 and COX-2 to their respective template sequences.** (A) Alignment of Human COX-1 [Uniprot ID: P23219] to the template sequence of *Ovis aries* [PDB ID: 1DIY: A], with 94% identity and 92% query coverage. (B) Sequence alignment of Human COX-2 [Uniprot ID: P35354] with the template sequence from *Mus musculus* [PDB ID: 1CVU: A], with an overall sequence identity of 88% and a query coverage of 91%. The binding cavity residues in both proteins were found to be conserved. The alignment diagram was generated using the MultAlign online interface.
(TIF)

Figure S2 **Intra-protein hydrogen bonding in COX-1 and COX-2 during model stabilisation MD run.** (A) Intra-protein H-bonds in COX-1, on an average COX-1 made around 390 intra-protein H-bonds over the entire MD simulation of 15ns. (B) Intra-protein H-bonds in COX-2, average H-bond number, for the entire course of 10ns MD run, was around 410 in COX-2. The plots have been generated using the GRACE plotting tool.
(TIF)

Figure S3 **3D orientation of amarogentin inside COX-1 and COX-2 binding site after docking.** (A) Amarogentin bound to the COX-1 active site shows two H-bonds, one with the amine group of Arg119 and the other with the hydroxyl side chains of Ser352. (B) Amarogentin makes five H-bonds with COX-2 after docking, two with the Arg106 and one each with Tyr371, Met508 and Ser516. The figure has been generated using Schrödinger Maestro open-source visualisation package.
(TIF)

Figure S4 **3D representation of the binding of amarogentin with two COX isoforms after 40ns time frame.** (A) Binding of amarogentin inside the binding cavity of COX-1, there is a shift in its orientation after 40ns and it forms only one H-bond with Ser352. (B) Binding pose of amarogentin inside the COX-2 binding cavity, the structure looks stable as it forms multiple H-bonds with the residues lining the cavity. The figure has been generated using Schrödinger Maestro open-source visualisation package.
(TIF)

Figure S5 **RMSD of amarogentin in complex with COX-1 and COX-2 over the entire 40ns simulation.** (A) Amarogentin shows a good overall stability after initial fluctuations inside the COX-1 binding cavity. (B) The stability of amarogentin is correlated with the overall stability of the amarogentin-COX-2 complex, with an increased stability in the final 10ns. The plot has been generated using the GRACE plotting tool.
(TIF)

Table S1 **PDB structures used to compare the docked poses.**
(DOC)

## References

[1] Vane JR (1971) Inhibition of prostaglandin synthesis as a mechanism of action for aspirin-like drugs. Nat New Biol 231: 232–235. 5284360 10.1038/newbio231232a0

[2] Ferreira SH, Moncada S, Vane JR (1971) Indomethacin and aspirin abolish prostaglandin release from the spleen. Nat New Biol 231: 237–239. 5284362 10.1038/newbio231237a0

[3] Smith JB, Willis AL (1971) Aspirin selectively inhibits prostaglandin production in human platelets. Nat New Biol 231: 235–237. 5284361 10.1038/newbio231235a0

[4] Vane JR (1978) The mode of action of aspirin-like drugs. Agents Actions 8: 430–431. 98977 10.1007/BF01968671

[5] Xie WL, Chipman JG, Robertson DL, Erikson RL, Simmons DL (1991) Expression of a mitogen-responsive gene encoding prostaglandin synthase is regulated by mRNA splicing. Proc Natl Acad Sci U S A 88: 2692–2696. 1849272 10.1073/pnas.88.7.2692 PMC51304

[6] Kujubu DA, Fletcher BS, Varnum BC, Lim RW, Herschman HR (1991) TIS10, a phorbol ester tumor promoter-inducible mRNA from Swiss 3T3 cells, encodes a novel prostaglandin synthase/cyclooxygenase homologue. J Biol Chem 266: 12866–12872. 1712772

[7] Warner TD, Giuliano F, Vojnovic I, Bukasa A, Mitchell JA, et al (1999) Nonsteroid drug selectivities for cyclo-oxygenase-1 rather than cyclo-oxygenase-2 are associated with human gastrointestinal toxicity: a full in vitro analysis. Proc Natl Acad Sci U S A 96: 7563–7568. 10377455 10.1073/pnas.96.13.7563 PMC22126

[8] Vane JR, Bakhle YS, Botting RM (1998) Cyclooxygenases 1 and 2. Annu Rev Pharmacol Toxicol 38: 97–120. 9597150 10.1146/annurev.pharmtox.38.1.97

[9] Chandrasekharan NV, Simmons DL (2004) The cyclooxygenases. Genome Biol 5: 241. 15345041 10.1186/gb-2004-5-9-241 PMC522864

[10] Kurumbail RG, Stevens AM, Gierse JK, McDonald JJ, Stegeman RA, et al (1996) Structural basis for selective inhibition of cyclooxygenase-2 by anti-inflammatory agents. Nature 384: 644–648. 8967954 10.1038/384644a0

[11] Claria J (2003) Cyclooxygenase-2 biology. Curr Pharm Des 9: 2177–2190. 14529398 10.2174/1381612033454054

[12] Cao Y, Prescott SM (2002) Many actions of cyclooxygenase-2 in cellular dynamics and in cancer. J Cell Physiol 190: 279–286. 11857443 10.1002/jcp.10068

[13] Kimura A, Tsuji S, Tsujii M, Sawaoka H, Iijima H, et al (2000) Expression of cyclooxygenase-2 and nitrotyrosine in human gastric mucosa before and after Helicobacter pylori eradication. Prostaglandins Leukot Essent Fatty Acids 63: 315–322. 11090259 10.1054/plef.2000.0220

[14] Kurumbail RG, Kiefer JR, Marnett LJ (2001) Cyclooxygenase enzymes: catalysis and inhibition. Curr Opin Struct Biol 11: 752–760. 11751058 10.1016/s0959-440x(01)00277-9

[15] Samad TA, Moore KA, Sapirstein A, Billet S, Allchorne A, et al (2001) Interleukin-1beta-mediated induction of Cox-2 in the CNS contributes to inflammatory pain hypersensitivity. Nature 410: 471–475. 11260714 10.1038/35068566

[16] Mouihate A, Clerget-Froidevaux MS, Nakamura K, Negishi M, Wallace JL, et al (2002) Suppression of fever at near term is associated with reduced COX-2 protein expression in rat hypothalamus. Am J Physiol Regul Integr Comp Physiol 283: R800–805. 12185016 10.1152/ajpregu.00258.2002

[17] Turini ME, DuBois RN (2002) Cyclooxygenase-2: a therapeutic target. Annu Rev Med 53: 35–57. 11818462 10.1146/annurev.med.53.082901.103952

[18] Langenbach R, Morham SG, Tiano HF, Loftin CD, Ghanayem BI, et al (1995) Prostaglandin synthase 1 gene disruption in mice reduces arachidonic acid-induced inflammation and indomethacin-induced gastric ulceration. Cell 83: 483–492. 8521478 10.1016/0092-8674(95)90126-4

[19] Zimmermann KC, Sarbia M, Schror K, Weber AA (1998) Constitutive cyclooxygenase-2 expression in healthy human and rabbit gastric mucosa. Mol Pharmacol 54: 536–540. 9730912 10.1124/mol.54.3.536

[20] Corley DA, Kerlikowske K, Verma R, Buffler P (2003) Protective association of aspirin/NSAIDs and esophageal cancer: a systematic review and meta-analysis. Gastroenterology 124: 47–56. 12512029 10.1053/gast.2003.50008

[21] Mahmud S, Franco E, Aprikian A (2004) Prostate cancer and use of nonsteroidal anti-inflammatory drugs: systematic review and meta-analysis. Br J Cancer 90: 93–99. 14710213 10.1038/sj.bjc.6601416 PMC2395299

[22] Wang WH, Huang JQ, Zheng GF, Lam SK, Karlberg J, et al (2003) Non-steroidal anti-inflammatory drug use and the risk of gastric cancer: a systematic review and meta-analysis. J Natl Cancer Inst 95: 1784–1791. 14652240 10.1093/jnci/djg106

[23] Garcia-Rodriguez LA, Huerta-Alvarez C (2001) Reduced risk of colorectal cancer among long-term users of aspirin and nonaspirin nonsteroidal antiinflammatory drugs. Epidemiology 12: 88–93. 11138826 10.1097/00001648-200101000-00015

[24] Subbaramaiah K, Dannenberg AJ (2003) Cyclooxygenase 2: a molecular target for cancer prevention and treatment. Trends Pharmacol Sci 24: 96–102. 12559775 10.1016/S0165-6147(02)00043-3

[25] Sheng H, Williams CS, Shao J, Liang P, DuBois RN, et al (1998) Induction of cyclooxygenase-2 by activated Ha-ras oncogene in Rat-1 fibroblasts and the role of mitogen-activated protein kinase pathway. J Biol Chem 273: 22120–22127. 9705357 10.1074/jbc.273.34.22120

[26] Mahdi JG, Mahdi AJ, Bowen ID (2006) The historical analysis of aspirin discovery, its relation to the willow tree and antiproliferative and anticancer potential. Cell Prolif 39: 147–155. 16542349 10.1111/j.1365-2184.2006.00377.x PMC6496865

[27] Vlot AC, Dempsey DA, Klessig DF (2009) Salicylic Acid, a multifaceted hormone to combat disease. Annu Rev Phytopathol 47: 177–206. 19400653 10.1146/annurev.phyto.050908.135202

[28] Vane JR, Botting RM (2003) The mechanism of action of aspirin. Thromb Res 110: 255–258. 14592543 10.1016/s0049-3848(03)00379-7

[29] Saklani A, Kutty SK (2008) Plant-derived compounds in clinical trials. Drug Discov Today 13: 161–171. 18275914 10.1016/j.drudis.2007.10.010

[30] Calixto JB, Otuki MF, Santos AR (2003) Anti-inflammatory compounds of plant origin. Part I. Action on arachidonic acid pathway, nitric oxide and nuclear factor kappa B (NF-kappaB). Planta Med 69: 973–983. 14735432 10.1055/s-2003-45141

[31] Surh YJ, Chun KS, Cha HH, Han SS, Keum YS, et al (2001) Molecular mechanisms underlying chemopreventive activities of anti-inflammatory phytochemicals: down-regulation of COX-2 and iNOS through suppression of NF-kappa B activation. Mutat Res 480−481: 243–268. 10.1016/s0027-5107(01)00183-x 11506818

[32] Dirsch VM, Vollmar AM (2001) Ajoene, a natural product with non-steroidal anti-inflammatory drug (NSAID)-like properties? Biochem Pharmacol 61: 587–593. 11239502 10.1016/s0006-2952(00)00580-3

[33] Brahmachari G, Mondal S, Gangopadhyay A, Gorai D, Mukhopadhyay B, et al (2004) Swertia (Gentianaceae): chemical and pharmacological aspects. Chem Biodivers 1: 1627–1651. 17191805 10.1002/cbdv.200490123

[34] Phoboo S, Pinto Mda S, Barbosa AC, Sarkar D, Bhowmik PC, et al (2013) Phenolic-linked biochemical rationale for the anti-diabetic properties of Swertia chirayita (Roxb. ex Flem.) Karst. Phytother Res 27: 227–235. 22523004 10.1002/ptr.4714

[35] Karan M, Vasisht K, Handa SS (1999) Antihepatotoxic activity of Swertia chirata on carbon tetrachloride induced hepatotoxicity in rats. Phytother Res 13: 24–30. 10189946 10.1002/(SICI)1099-1573(199902)13:1<24::AID-PTR378>3.0.CO;2-L

[36] Karan M, Vasisht K, Handa SS (1999) Antihepatotoxic activity of Swertia chirata on paracetamol and galactosamine induced hepatotoxicity in rats. Phytother Res 13: 95–101. 10190179 10.1002/(SICI)1099-1573(199903)13:2<95::AID-PTR379>3.0.CO;2-4

[37] Saha P, Mandal S, Das A, Das PC, Das S (2004) Evaluation of the anticarcinogenic activity of Swertia chirata Buch.Ham, an Indian medicinal plant, on DMBA-induced mouse skin carcinogenesis model. Phytother Res 18: 373–378. 15173996 10.1002/ptr.1436

[38] Ray S, Majumder HK, Chakravarty AK, Mukhopadhyay S, Gil RR, et al (1996) Amarogentin, a naturally occurring secoiridoid glycoside and a newly recognized inhibitor of topoisomerase I from Leishmania donovani. J Nat Prod 59: 27–29. 8984149 10.1021/np960018g

[39] Behrens M, Brockhoff A, Batram C, Kuhn C, Appendino G, et al (2009) The human bitter taste receptor hTAS2R50 is activated by the two natural bitter terpenoids andrographolide and amarogentin. J Agric Food Chem 57: 9860–9866. 19817411 10.1021/jf9014334

[40] Medda S, Mukhopadhyay S, Basu MK (1999) Evaluation of the in-vivo activity and toxicity of amarogentin, an antileishmanial agent, in both liposomal and niosomal forms. J Antimicrob Chemother 44: 791–794. 10590280 10.1093/jac/44.6.791

[41] Pal D, Sur S, Mandal S, Das A, Roy A, et al (2012) Prevention of liver carcinogenesis by amarogentin through modulation of G1/S cell cycle check point and induction of apoptosis. Carcinogenesis 33: 2424–2431. 22948180 10.1093/carcin/bgs276

[42] Saha P, Mandal S, Das A, Das S (2006) Amarogentin can reduce hyperproliferation by downregulation of Cox-II and upregulation of apoptosis in mouse skin carcinogenesis model. Cancer Lett 244: 252–259. 16517061 10.1016/j.canlet.2005.12.036

[43] Update on activities at the Universal Protein Resource (UniProt) in 2013. Nucleic Acids Res 41: D43–47. 23161681 10.1093/nar/gks1068 PMC3531094

[44] Altschul SF, Gish W, Miller W, Myers EW, Lipman DJ (1990) Basic local alignment search tool. J Mol Biol 215: 403–410. 2231712 10.1016/S0022-2836(05)80360-2

[45] Bernstein FC, Koetzle TF, Williams GJ, Meyer EF Jr, Brice MD, et al (1977) The Protein Data Bank: a computer-based archival file for macromolecular structures. J Mol Biol 112: 535–542. 875032 10.1016/s0022-2836(77)80200-3

[46] Corpet F (1988) Multiple sequence alignment with hierarchical clustering. Nucleic Acids Res 16: 10881–10890. 2849754 10.1093/nar/16.22.10881 PMC338945

[47] Eswar N, Eramian D, Webb B, Shen MY, Sali A (2008) Protein structure modeling with MODELLER. Methods Mol Biol 426: 145–159. 18542861 10.1007/978-1-60327-058-8_8

[48] Eisenberg D, Luthy R, Bowie JU (1997) VERIFY3D: assessment of protein models with three-dimensional profiles. Methods Enzymol 277: 396–404. 9379925 10.1016/s0076-6879(97)77022-8

[49] Colovos C, Yeates TO (1993) Verification of protein structures: patterns of nonbonded atomic interactions. Protein Sci 2: 1511–1519. 8401235 10.1002/pro.5560020916 PMC2142462

[50] Pronk S, Pall S, Schulz R, Larsson P, Bjelkmar P, et al (2013) GROMACS 4.5: a high-throughput and highly parallel open source molecular simulation toolkit. Bioinformatics 29: 845–854. 23407358 10.1093/bioinformatics/btt055 PMC3605599

[51] Schuttelkopf AW, van Aalten DM (2004) PRODRG: a tool for high-throughput crystallography of protein-ligand complexes. Acta Crystallogr D Biol Crystallogr 60: 1355–1363. 15272157 10.1107/S0907444904011679

[52] Alonso H, Bliznyuk AA, Gready JE (2006) Combining docking and molecular dynamic simulations in drug design. Med Res Rev 26: 531–568. 16758486 10.1002/med.20067

[53] Morris GM, Huey R, Lindstrom W, Sanner MF, Belew RK, et al (2009) AutoDock4 and AutoDockTools4: Automated docking with selective receptor flexibility. J Comput Chem 30: 2785–2791. 19399780 10.1002/jcc.21256 PMC2760638

[54] Pence HE, Williams A (2010) ChemSpider: an online chemical information resource. Journal of Chemical Education 87: 1123–1124.

[55] Massova I, Kollman PA (2000) Combined molecular mechanical and continuum solvent approach (MM-PBSA/GBSA) to predict ligand binding. Perspectives in drug discovery and design 18: 113–135.

[56] Kollman PA, Massova I, Reyes C, Kuhn B, Huo S, et al (2000) Calculating structures and free energies of complex molecules: combining molecular mechanics and continuum models. Acc Chem Res 33: 889–897. 11123888 10.1021/ar000033j

[57] Campanera JM, Pouplana R (2010) MMPBSA decomposition of the binding energy throughout a molecular dynamics simulation of amyloid-beta (Abeta(10-35)) aggregation. Molecules 15: 2730–2748. 20428075 10.3390/molecules15042730 PMC6257327

[58] Kuhn B, Kollman PA (2000) Binding of a diverse set of ligands to avidin and streptavidin: an accurate quantitative prediction of their relative affinities by a combination of molecular mechanics and continuum solvent models. J Med Chem 43: 3786–3791. 11020294 10.1021/jm000241h

[59] Gilson MK, Zhou HX (2007) Calculation of protein-ligand binding affinities. Annu Rev Biophys Biomol Struct 36: 21–42. 17201676 10.1146/annurev.biophys.36.040306.132550

[60] Sitkoff D, Sharp KA, Honig B (1994) Accurate calculation of hydration free energies using macroscopic solvent models. The Journal of Physical Chemistry 98: 1978–1988.

[61] Bashford D, Case DA (2000) Generalized born models of macromolecular solvation effects. Annu Rev Phys Chem 51: 129–152. 11031278 10.1146/annurev.physchem.51.1.129

[62] Weiser J, Shenkin PS, Still WC (1999) Approximate atomic surfaces from linear combinations of pairwise overlaps (LCPO). Journal of Computational Chemistry 20: 217–230.

[63] Plount Price ML, Jorgensen WL (2000) Analysis of binding affinities for celecoxib analogues with COX-1 and COX-2 from combined docking and Monte Carlo simulations and insight into the COX-2/COX-1 selectivity. Journal of the American Chemical Society 122: 9455–9466.

[64] Kiefer JR, Pawlitz JL, Moreland KT, Stegeman RA, Hood WF, et al (2000) Structural insights into the stereochemistry of the cyclooxygenase reaction. Nature 405: 97–101. 10811226 10.1038/35011103

[65] Adeniyi AA, Ajibade PA (2013) Comparing the Suitability of Autodock, Gold and Glide for the Docking and Predicting the Possible Targets of Ru (II)-Based Complexes as Anticancer Agents. Molecules 18: 3760–3778. 23529035 10.3390/molecules18043760 PMC6270031

[66] Hou T, Wang J, Li Y, Wang W (2011) Assessing the performance of the MM/PBSA and MM/GBSA methods. 1. The accuracy of binding free energy calculations based on molecular dynamics simulations. J Chem Inf Model 51: 69–82. 21117705 10.1021/ci100275a PMC3029230

[67] Riendeau D, Percival M, Brideau C, Charleson S, Dube D, et al (2001) Etoricoxib (MK-0663): preclinical profile and comparison with other agents that selectively inhibit cyclooxygenase-2. Journal of Pharmacology and Experimental Therapeutics 296: 558–566. 11160644

