# Supplementary figures and images for: The Bitter Barricading of Prostaglandin Biosynthesis Pathway: Understanding the Molecular Mechanism of Selective Cyclooxygenase-2 Inhibition by Amarogentin, a Secoiridoid Glycoside from Swertia chirayita
Source: PLoS One. 2014 Mar 6;9(3):e90637. doi: 10.1371/journal.pone.0090637 (PMC3946170; doi:10.1371/journal.pone.0090637)

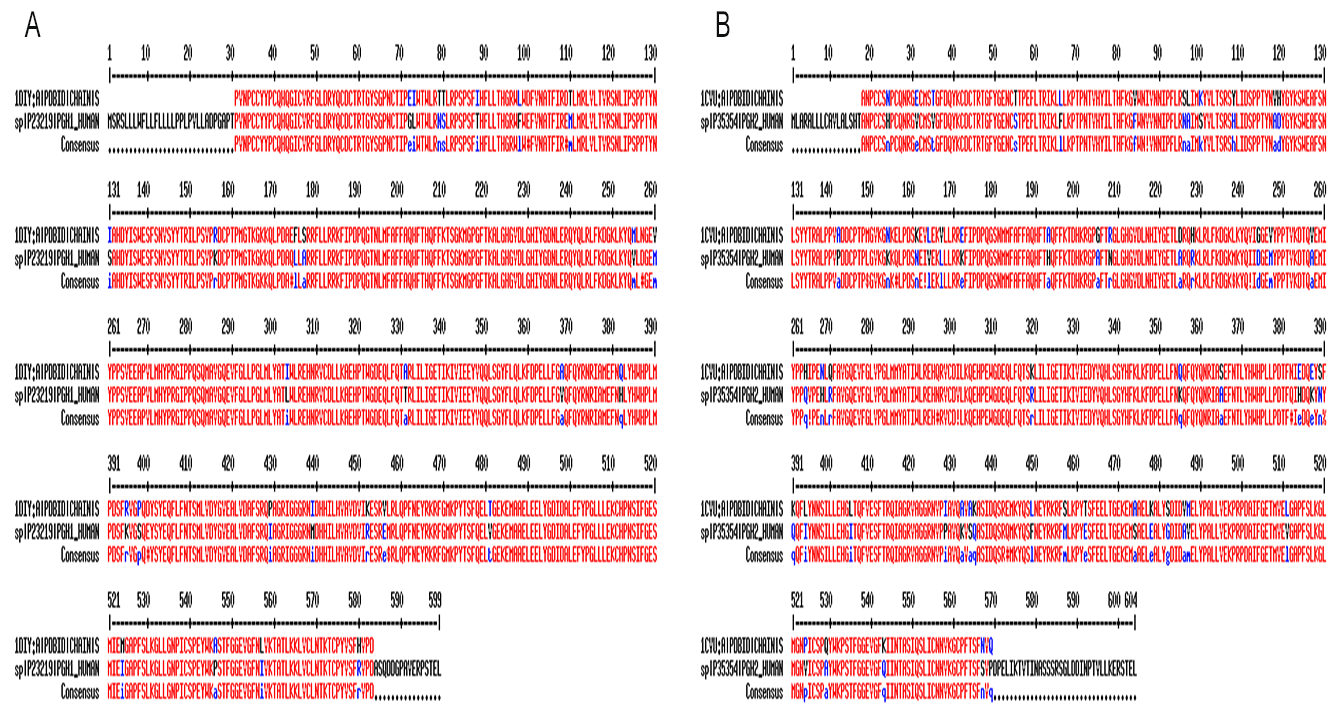

Supplement: Figure S1 — Pairwise sequence alignment of COX-1 and COX-2 to their respective template sequences. (A) Alignment of Human COX-1 [Uniprot ID: P23219] to the template sequence of Ovis aries [PDB ID: 1DIY: A], with 94% identity and 92% query coverage. (B) Sequence alignment of Human COX-2 [Uniprot ID: P35354] with the template sequence from Mus musculus [PDB ID: 1CVU: A], with an overall sequence identity of 88% and a query coverage of 91%. The binding cavity residues in both proteins were found to be conserved. The alignment diagram was generated using the MultAlign online interface. (TIF) [file pone.0090637.s001.tif]

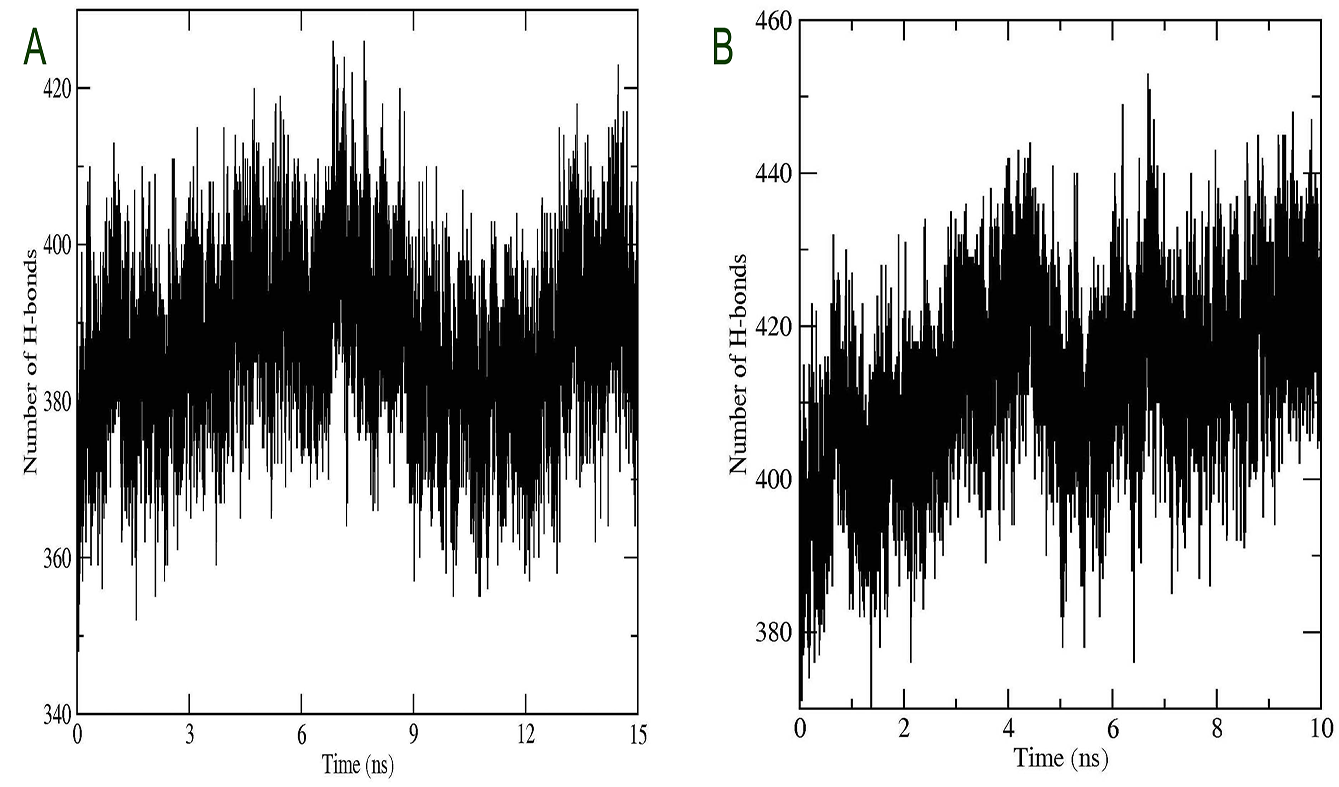

Supplement: Figure S2 — Intra-protein hydrogen bonding in COX-1 and COX-2 during model stabilisation MD run. (A) Intra-protein H-bonds in COX-1, on an average COX-1 made around 390 intra-protein H-bonds over the entire MD simulation of 15ns. (B) Intra-protein H-bonds in COX-2, average H-bond number, for the entire course of 10ns MD run, was around 410 in COX-2. The plots have been generated using the GRACE plotting tool. (TIF) [file pone.0090637.s002.tif]

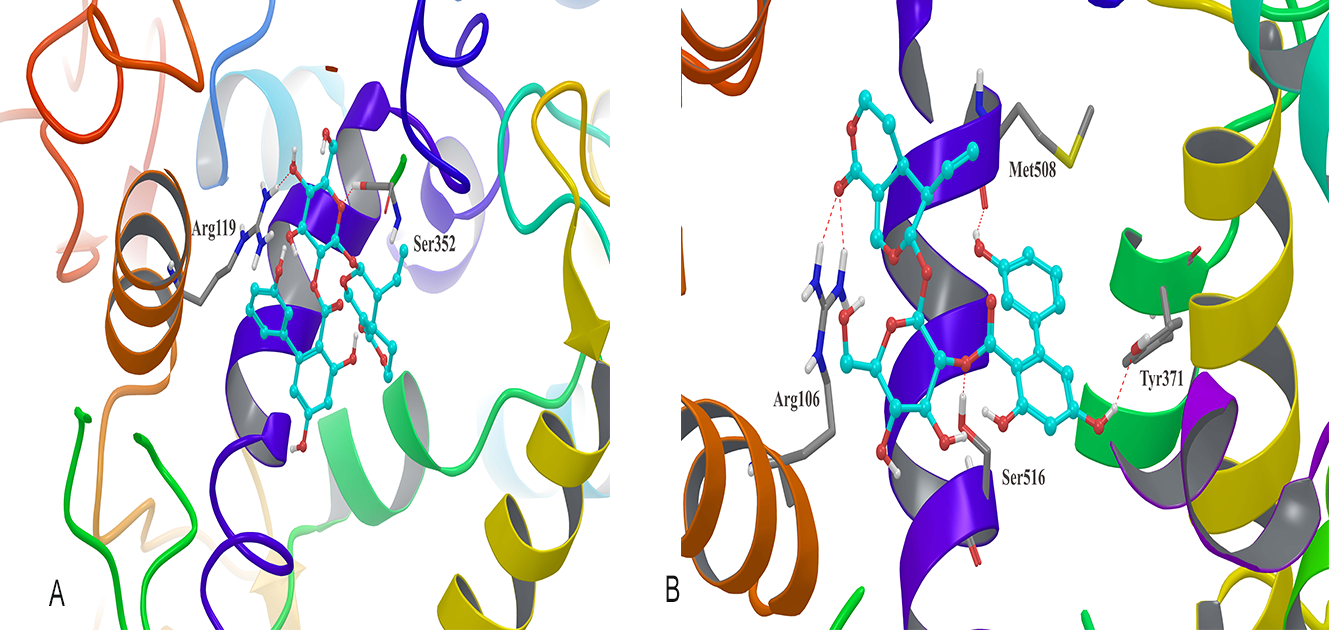

Supplement: Figure S3 — 3D orientation of amarogentin inside COX-1 and COX-2 binding site after docking. (A) Amarogentin bound to the COX-1 active site shows two H-bonds, one with the amine group of Arg119 and the other with the hydroxyl side chains of Ser352. (B) Amarogentin makes five H-bonds with COX-2 after docking, two with the Arg106 and one each with Tyr371, Met508 and Ser516. The figure has been generated using Schrödinger Maestro open-source visualisation package. (TIF) [file pone.0090637.s003.tif]

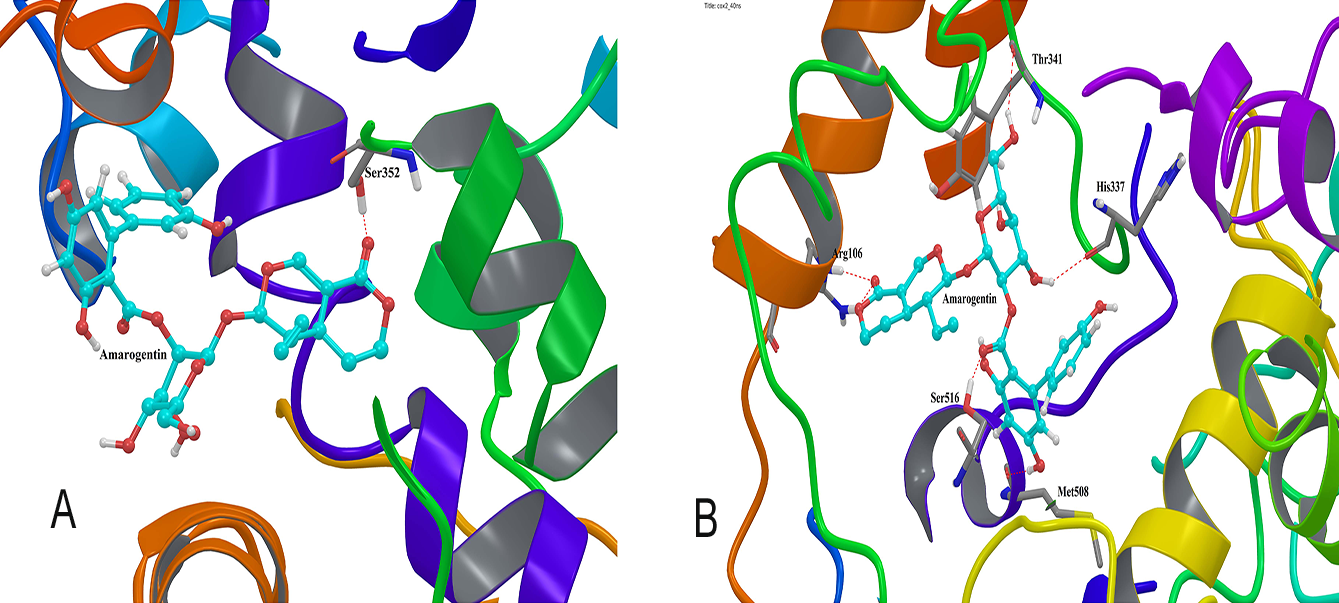

Supplement: Figure S4 — 3D representation of the binding of amarogentin with two COX isoforms after 40ns time frame. (A) Binding of amarogentin inside the binding cavity of COX-1, there is a shift in its orientation after 40ns and it forms only one H-bond with Ser352. (B) Binding pose of amarogentin inside the COX-2 binding cavity, the structure looks stable as it forms multiple H-bonds with the residues lining the cavity. The figure has been generated using Schrödinger Maestro open-source visualisation package. (TIF) [file pone.0090637.s004.tif]

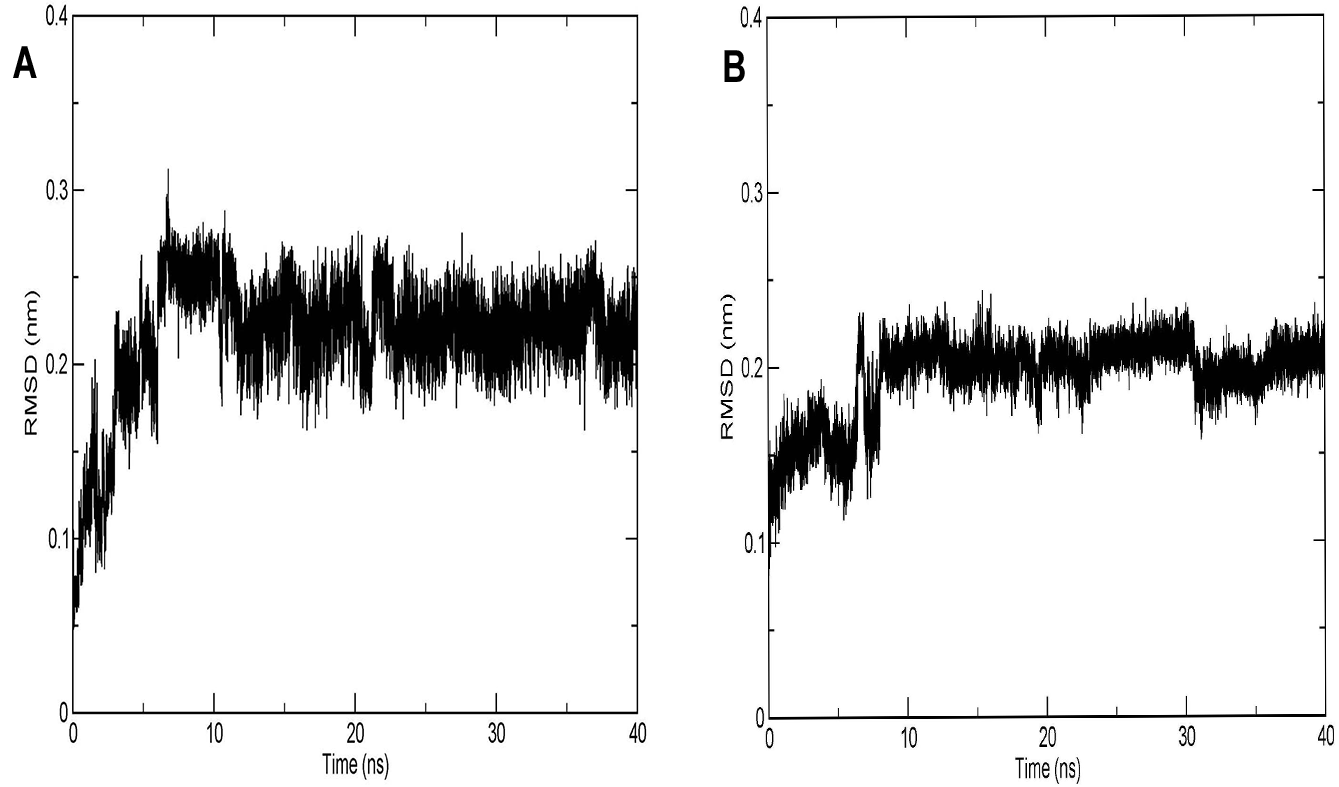

Supplement: Figure S5 — RMSD of amarogentin in complex with COX-1 and COX-2 over the entire 40ns simulation. (A) Amarogentin shows a good overall stability after initial fluctuations inside the COX-1 binding cavity. (B) The stability of amarogentin is correlated with the overall stability of the amarogentin-COX-2 complex, with an increased stability in the final 10ns. The plot has been generated using the GRACE plotting tool. (TIF) [file pone.0090637.s005.tif]
