# Supplementary material for: The Bitter Barricading of Prostaglandin Biosynthesis Pathway: Understanding the Molecular Mechanism of Selective Cyclooxygenase-2 Inhibition by Amarogentin, a Secoiridoid Glycoside from Swertia chirayita
Source: PLoS One. 2014 Mar 6;9(3):e90637. doi: 10.1371/journal.pone.0090637 (PMC3946170; doi:10.1371/journal.pone.0090637)
Supplement: Table S1 — PDB structures used to compare the docked poses. (DOC) [file pone.0090637.s006.doc]

**Supplementary Information 3: PDB structures used to compare the docked poses.**

|  | **COX-1** | | | | **COX-2** | | | |
| --- | --- | --- | --- | --- | --- | --- | --- | --- |
| **Compound** | **PDB id** | **H bonds** | **Resolution** | **Organism** | **PDB id** | **H bonds** | **Resolution** | **Organism** |
| Flurbiprofen | 3N8Z | Tyr355, Arg 120 | 2.9 | *Ovis aries* | 3PGH | Tyr355, Arg 120 | 2.5 | *Mus musculus* |
| Indometacine | 2OYU | Ser353, Leu352 | 2.7 | *Ovis aries* | 4COX | Tyr355 | 2.9 | *Mus musculus* |
| Diclofenac | 3N8Y | Tyr385, Ser530 | 2.6 | *Ovis aries* | 1PXX | Tyr385, Ser530 | 2.9 | *Mus musculus* |
| Naproxen |  |  |  |  | 3NT1 | Tyr355, Arg 120 | 1.73 | *Mus musculus* |
| Celecoxib | 3KK6 | Gln192, Leu352 | 2.75 | *Ovis aries* | 3LN1 | Arg499, Gln178, Ser339,  Leu338 | 2.4 | *Mus musculus* |
| Nimesulide | 3N8X | Arg120 | 3.75 | *Ovis aries* |  |  |  |  |

A total of 10 PDB entries of co-crystallised drug molecules, five each for COX-1 and COX-2, were retrieved. The H-bonding pattern of the PDB entries was compared to the obtained docking results of these inhibitors.
